# Global analysis of HLA-A2 restricted MAGE-A3 tumor antigen epitopes and corresponding TCRs in non-small cell lung cancer

**DOI:** 10.7150/thno.84710

**Published:** 2023-08-06

**Authors:** Bei Zhang, Zhiyao Ren, Jianfu Zhao, Yue Zhu, Boya Huang, Chanchan Xiao, Yan Zhang, Jieping Deng, Lipeng Mao, Lei Tang, Dan Lan, Lijuan Gao, Hongyi Zhang, Guobing Chen, Oscar Junhong Luo

**Affiliations:** 1 Department of Systems Biomedical Sciences, School of Medicine, Jinan University, Guangzhou, China.; 2 Guangdong-Hong Kong-Macau Great Bay Area Geroscience Joint Laboratory, School of Medicine, Jinan University, Guangzhou, China.; 3 Guangzhou Geriatric Hospital, Guangzhou, China.; 4 Collaborative Innovation Center for Civil Affairs of Guangzhou, Guangzhou, China.; 5 Department of Oncology, Research Center of Cancer Diagnosis and Therapy, the First Affiliated Hospital, Jinan University, Guangzhou, China.; 6 Department of Microbiology and Immunology; Institute of Geriatric Immunology; School of Medicine, Jinan University, Guangzhou, China.; 7 School of Life Science & Technology, China Pharmaceutical University, Nanjing, China.

**Keywords:** NSCLC, MAGE-A3, Epitope-specific TCR, Cancer immunotherapy

## Abstract

**Background:** Advanced non-small cell lung cancer (NSCLC) is the most common type of lung cancer with poor prognosis. Adoptive cell therapy using engineered T-cell receptors (TCRs) targeting cancer-testis antigens, such as Melanoma-associated antigen 3 (MAGE-A3), is a potential approach for the treatment of NSCLC. However, systematic analysis of T cell immune responses to MAGE-A3 antigen and corresponding antigen-specific TCR is still lacking.

**Methods:** In this study, we comprehensively screened HLA-A2 restricted MAGE-A3 tumor epitopes and characterized the corresponding TCRs using *in vitro* artificial antigen presentation cells (APC) system, single-cell transcriptome and TCR V(D)J sequencing, and machine-learning. Furthermore, the tumor-reactive TCRs with killing potency was screened and verified.

**Results:** We identified the HLA-A2 restricted T cell epitopes from MAGE-A3 that could effectively induce the activation and cytotoxicity of CD8+ T cells using artificial APC* in vitro*. A cohort of HLA-A2+ NSCLC donors demonstrated that the number of epitope specific CD8+ T cells increased in NSCLC than healthy controls when measured with tetramer derived from the candidate MAGE-A3 epitopes, especially epitope Mp4 (MAGE-A3: 160-169, LVFGIELMEV). Statistical and machine-learning based analyses demonstrated that the MAGE-A3-Mp4 epitope-specific CD8+ T cell clones were mostly in effector and proliferating state. Importantly, T cells artificially expressing the MAGE-A3-Mp4 specific TCRs exhibited strong MAGE-A3+ tumor cell recognition and killing effect. Cross-reactivity risk analysis of the candidates TCRs showed high binding stability to MAGE-A3-Mp4 epitope and low risk of cross-reaction.

**Conclusions:** This work identified candidate TCRs potentially suitable for TCR-T design targeting HLA-A2 restricted MAGE-A3 tumor antigen.

## Introduction

Lung cancer is one of the primary causes of cancer-related death worldwide 1. Non-small cell lung cancer (NSCLC), the most common subtype, comprised approximately 85% of the newly diagnosed lung cancer cases 2. Despite the recent advances in the treatment of NSCLC, including surgery, chemotherapy, radiotherapy and targeted therapy, there has been little improvement in overall patient survival 3. Although immune checkpoint blockade (ICB) reinvigorates anti-tumor immune responses by disrupting co-inhibitory T-cell signaling, relapse frequently occurs after ICB treatment and acquired resistance often emergence after initial response 4. Novel immunotherapy strategy and therapeutic target has become a recent research focus in the effort of finding cures for NSCLC.

NSCLC is characterized with high expression of cancer-testis antigens (CTAs) 5, 6, whose expression in healthy tissue is restricted to immune-privileged sites, and have been considered as ideal targets for immunotherapy 7. Thus, current T cell therapies mainly focus on CTAs including New York esophageal squamous cell carcinoma-1 (NYESO1) 8 and Melanoma-associated antigens (MAGEs) 9, 10. Melanoma-associated antigen 3 (MAGE-A3), a CTA highly expressed in NSCLC 11, 12, is intracellularly processed with human leukocyte antigens (HLAs) on the cell surface, therefore, was utilized as an important therapeutic target in NSCLC. However, MAGE-A3 based antibody therapy and tumor vaccines showed limited efficacy. Adjuvant treatment with the MAGE-A3 immunotherapeutic did not increase disease-free survival in patients with MAGE-A3-positive surgically resected NSCLC despite the production of antibodies 13.

T cells play an essential role in the antitumor immunity by directly killing tumor cells. With durable cancer responses, adoptive T cell transfer with a tumor antigen-specific T-cell receptor (TCR) is currently an effective approach to cancer treatment 14-16. However, the major challenge in TCR-engineered T cell (TCR-T) immunotherapy is the discovery of natural TCRs with high activity and specificity to tumor antigens 17, 18. On one hand, most endogenous tumor-specific TCRs elicit weak functional responses because some epitopes are weakly recognized by low-affinity natural TCRs. On the other hand, synthetic TCRs for increased tumor antigen recognition is complicated due to the risk of introducing cross-reactions 18. TCR-T targeting MAGE-A3 has reported lethal off-target cross-reactivity in clinical trials because of the high affinity of the TCR combined with the tumor pMHC (peptide-major histocompatibility complex) 19-21. Thus, it is urgent to screen MAGE-A3 epitopes inducing T cell immune response and the corresponding TCRs with appropriate affinity.

Currently, systematic analysis of T cell immune responses to MAGE-A3 antigen and corresponding antigen-specific TCR is still lacking. In this study, we identified HLA-A2 restricted T cell epitopes from MAGE-A3 using *in vitro* artificial antigen-presenting cells (APCs). Then, an HLA-A2+ NSCLC cohort was recruited and used to verify the candidate MAGE-A3 epitopes. In the process, we identified the MAGE-A3-specific TCR repertoire by single-cell transcriptomic analysis (scRNA-seq) and TCR sequencing (scTCR-seq), and systematically screened for candidate MAGE-A3 antigen-specific TCRs with high specificity by co-considering T cell cellular phenotype and clonotype expansion. Finally, we verified that T cells with the candidate TCRs could respond to tumor cells expressing MAGE-A3 with strong killing effect *in vitro*. We also discovered lung cancer patients with TCR repertoire that were more similar to the candidate TCRs would have better response to ICB therapy. We concluded that the identified MAGE-A3-specific TCRs could be used for personalized TCR-T cell therapy design and prediction of immunotherapy response in NSCLC.

## Materials and Methods

### Human samples

This study was approved by the Institutional Review Board of the School of Medicine of Jinan University (JNUKY-2021-009 and JNUKY-2022-101). Peripheral blood samples (approximately 10 mL per sample) were extracted from NSCLC patients diagnosed at the First Affiliated Hospital of Jinan University (Table S1). The peripheral blood from the healthy donors were collected at Guangzhou Blood Center (200-400 mL per sample). Peripheral blood mononuclear cells (PBMCs) were isolated by density gradient centrifugation using lymphocyte separation medium (GE, US) and stained with PE conjugated anti-human HLA-A2 antibody (BioLegend, Cat#343305, US) to identify the HLA-A2+ donors.

### Immunohistochemistry of MAGE-A3

Anti-MAGE-A3 antibody was stained at 1:150 (abcam, ab223162), followed by HRP-conjugated secondary antibody. The slice was counterstained with hematoxylin. Automated imaging of the tissue microarray (Table S2 and Table S3) was performed using digital scanning (Pannoramic MIDI, 3DHISTECH). The tissue microarray of lung adenocarcinoma (Outdo Biotech, Cat#HLugA150CS03) and lung squamous cell carcinoma (Outdo Biotech, Cat#Hlug-Squ150CS-01) consisted of 75 primary tumors and 75 paracancerous tissues, respectively. Human NSCLC specimens were scored in a semi-quantitative manner due to the heterogeneity of the staining of MAGE-A3 proteins. The staining percentage was scored as follows: ≤5% = 0, 5-30% = 1, 30-70% = 2, and ≥ 70% = 3. The staining intensity was categorized as follows: none = 0, weak = 1, moderate = 2, and strong = 3. A final immunoreactivity scores (IRS) was obtained for each case as the sum of the percentage and the intensity scores.

### T cell epitopes prediction

The protein amino acid sequence of MAGE-A3 (NP_005353.1) was downloaded from the GenBank Database (https://www.ncbi.nlm.nih.gov/genbank/) and used for CD8 T cell epitope prediction with the MHC-I binding tool (http://tools.iedb.org/mhci). The prediction method used was IEDB Recommended 2.22 (NetMHCpan EL), with MHC allele selected as HLA-A2, which is the most frequent class I HLA genotype among Chinese population 22, 23. Peptide containing 9-10 amino acids, with the best prediction score and low IC_50_ was used as the candidate epitopes. Finally, 10 candidate epitopes were predicted and synthesized by GenScript Biotechnology Co., Ltd (Nanjing, China) with purity > 98% for downstream experiments.

### Peptides binding affinity on T2 cells

The T2 cells expressing HLA-A2 molecules on the cell surface were used as artificial APCs. Thus, the peptide binding affinity on T2 cells was examined. The 10 predicted epitope peptides were dissolved in DMSO at 10 mM stock concentration. Then, the titration of peptide concentration was performed as described previously 24. The T2 cell line was shared by Anna Gil (University of Massachusetts Medical School), which is TAP-deficient T cell expressing HLA-A2 protein on the cell surface 25. T2 cells (0.2×10^6^/well) were seeded into 96-well plates and incubated with 20 µM peptides at 37 °C for 4 hours. DMSO was set as blank control, the HLA-A2 restricted Influenza A (flu) M1 peptide (M58-66 GILGFVFTL) was set as positive control and HLA-A11 restricted EBNA3B (EBV) virus peptide (E416-424 IVTDFSVIK) was set as negative control 26-28. Cells were stained with anti-HLA-A2 antibody (BioLegend, Cat#343305, US) and acquired using flow cytometer FACS Canto (BD).

### HLA-A2 peptide exchange ELISA

Recombinant HLA-A2 monomer with UV exchangeable peptide were purchased commercially (BioLegend, Cat#280003, US). Then, 20 µL diluted peptide (400 µM) in PBS and 20 µL Flex-T™ monomer (200 µg/mL) were added into a 96-well U-bottom plate. The UV peptide exchange was performed over 30 min using Triple-Purpose UV Analyzer ZF-1 at 365 nm. The Flex-T™ Human Class I Peptide Exchange ELISA Kit (BioLegend Cat#447207, US) was used to evaluate UV activated peptide exchange efficiency according to the manufacturer's instructions. DMSO was set as UV control, flu M1 peptide (M58-66, GILGFVFTL) was set as positive control, and EBV virus peptide (E416-424 IVTDFSVIK) was set as negative control.

### FACS sorting of antigen-specific CD8+ T cells

Streptavidin (BioLegend Cat#405203, US) conjugated fluorophore was added in the peptide-exchanged monomer formed in the above steps for a final 30:3.3 molar ratio of MHC:streptavidin and incubated on ice in the dark for 30 min. Then, 2.4 µL blocking solution containing 1.6 µL 50 mM biotin (Thermo Fisher, Cat#B20656, US) and 198.4 µL PBS was added and incubated overnight at 4-8 °C. Tetramer staining was performed in 1% BSA in Fc Blocking solution (Biolegend, Cat#422302, US) at room temperature for 1 h and then stained with human CD8 antibody (BioLegend Cat#344721, US) for 30 min at 4 °C. Then the flow cytometer FACSAria III (BD) was used to sort antigen-specific CD8+ T cells.

### Activation and cytotoxicity analysis of CD8+ T cells

On day 0, T2 cells were treated with 20 µg/mL mitomycin C (Sigma, Cat#50-07-07) for 30 min and loaded with MAGE-A3 epitopes (Mp1, Mp2, Mp3, Mp4, Mp5, Mp6, Mp7, Mp8, Mp9 and Mp10, respectively) or flu M1 peptide (M58-66, GILGFVFTL) as positive control and EBV virus peptide (E416-424 IVTDFSVIK) as negative control for 4 hours. T2 cells loaded with MAGE-A3 epitopes were pooled for downstream experiments. CFSE was used to detect whether T2 cells proliferate or not (Figure S1A). CD8+ T cells were isolated from PBMC by EasySep Negative selection Kit (STEMCELL, Cat#17953, Canada) and co-cultivated with peptide-loaded T2 cells at a 1:1 ratio (0.2×10^6^: 0.2×10^6^) in a 96-well plate for 7 days 24, providing the first signal to T cell activation. The cells were then stimulated with 1 µg/mL anti-human CD28 (BioLegend, Cat#302901, US) and 50 IU/mL IL-2 (SL PHARM, Recombinant Human Interleukin-2(125Ala) Injection) for 7 days to provide the second signal and co-stimulatory signals for T cell activation. Subsequently, the T cell activation marker CD69 (BioLegend, Cat#310909, US) and CD137 (BioLegend, Cat#309809, US) were analyzed at 16 hours. On day 0 and day 7, tetramer specific CD8+ T cells were detected. In addition, cells were re-stimulated with peptides for 5 hours in the presence of Leuko Act Cktl with GolgiPlug (BD, Cat#550583, US) and 50 IU/mL IL-2 on day 7, then the production of IFN-γ and Granzyme B (GZMB) was checked with anti-human IFN-γ antibody (BioLegend, Cat#502524, US) and anti-human GZMB antibody (BioLegend, Cat#515403, US).

### Co-culture and ELISpot

PBMCs were cultured in RPMI 1640 media supplemented with 10% fetal bovine serum (LONSERA, Cat#S711-001S, Uruguay) overnight after resuscitation. Co-culture for IFN-γ ELISpot assay was set up using 1×10^5^ peptide loading T2 cells and 1×10^5^ PBMCs in the 96-well format. Anti-CD3 mAb CD3-2 was used as the positive stimulant. Cell suspensions were transferred to precoated human IFN-γ ELISpot Plus kits (MabTech, Cat#3420-4HPW-2, US) for 2 days according to the manufacturer's instructions. Spots were imaged and counted by ELISpot reader (Mabtech).

### Single-cell RNA and TCR sequencing

The epitope-specific CD8+ T cells were sorted by flow cytometer FACSAria III (BD) using the PE conjugated tetramers and APC conjugated anti-CD8 antibody. Cell number and viability were checked after surface protein hashtag staining (Hashtag_3: BioLegend, Cat#394665, US; Hashtag_6: BioLegend, Cat#394671, US). Then droplet-encapsulation single-cell sequencing experiments were performed, and 10,000 living single cells were loaded onto Chromium Controller (10x Genomics) for each run. After droplet-encapsulation, single-cell cDNA synthesis and amplification, sequencing libraries were generated using Chromium Single Cell 5' Feature Barcode Library Kit (10x Genomics), Chromium Single Cell 5' Library & Gel Bead Kit (10x Genomics), and Chromium Single Cell V(D)J Enrichment Kit (Human T Cell, 10x Genomics) according to the manufacturer's instructions. The libraries from each loaded channel (up to eight channels) were multiplexed together and sequenced on an Illumina Novaseq 6000.

### Single-cell sequencing data processing

The 10x Genomics Cell Ranger software (v6.1.0) was used to process the fastq files with Human reference GRCh38-2020-A (https://support.10xgenomics.com/single-cell-gene-expression/software/release-notes/build) for the scRNA-seq and hashtag antibodies sequencing, with default parameter settings. The result files were directly loaded into R package Seurat (v4.0.4) for further analysis. Cells with nFeature_RNA > 200 and nFeature_RNA < 6000, as well as the percent of reads mapped to mitochondria genes < 10%, were kept for FindVariableFeatures to extract top 2,000 variable genes for subsequent analysis. The highest normalized hashtag count value was chosen to assign each cell to the corresponding sample. FindClusters (resolution = 1.2) was used to divide the cells into 15 clusters with the first 10 principal components chosen from PCA analysis. The top 10 marker genes for each cluster were identified by FindAllMarkers (Table S4). AddModuleScore was used to calculate score for assigned gene set (Table **S5**) and repOverlap from R package immunarch (v0.6.6) was used to aggregate the shared TCR clonotypes between different cluster.

### Prediction of antigen specific TCRs

Tessa 29 (https://github.com/jcao89757/tessa) is a model to quantitatively interpret the functional relevance of T cell repertoire, which identifies TCR clonotypes in the same network having similar functions and may be specific to the same antigen. The input files of tessa are scRNA-seq expression and scTCR-seq derived TCR CDR3β data matched through cell barcode. We used tessa to identify TCR networks and corresponding center TCR from each network using the MAGE-A3-Mp4 epitope-specific scRNA-seq and scTCR-seq data described above. Other parameters were set as default.

pMTnet 30 (https://github.com/tianshilu/pMTnet) is a TCR repertoire analysis machine-learning algorithm, which can be used for predicting and ranking binding affinity between TCR and pMHC. pMTnet was used to identify and rank the MAGE-A3-Mp4 epitope-specific TCRs derived from the single-cell sequencing data described above. The input files of pMTnet are TCR CDR3β sequence, epitope sequence as well as HLA allele information. Other parameters were set as default. TCR Binding Rank to pMHC was output as a final result. The output of pMTnet is a continuous variable ranging between 0 and 1, a smaller rank value indicates a more stable binding. The rank values of each TCRs were used as a reference for their binding affinity to MAGE-A3-Mp4 pMHC.

FuzzyWuzzy (https://github.com/seatgeek/fuzzywuzzy), a python package for string pattern-matching, was used to compare the TCR CDR3β sequences of NSCLC patients before and after ICB therapy to the MAGE-A3 antigen-specific TCRs identified in this study. Optional parameters were set as default. 80% similarity was used as a sequence match threshold for TCR CDR3β sequence comparison.

### Western blot

Cells were lysed with RIPA buffer (Beyotime, P0013B) for total lysate extraction. Then, 10 µg protein was used for electrophoresis with 10% SDS-PAGE and then transferred to a polyvinylidene difluoride membrane. The membrane was first blocked by 5% BSA for 1.5 h at room temperature, followed by incubation at 4 °C overnight with the following primary antibodies: MAGE-A3 (abcam, ab223162) and GAPDH (Proteintech, HRP-60004). After washing three times with TBST, the membrane was incubated with anti-mouse and anti-rabbit horseradish peroxidase conjugated secondary antibodies (boster, BA1054 and BA1051) for 2 h at room temperature.

### Generation of TCR-T J76 cells

The α and β nucleotide sequences of a sequenced TCR were linked by the 2A sequence (gtgaaacagactttgaattttgaccttctcaagttggcgggagacgtggagtccaacccagggccc), and then synthesized and subcloned into the 18121 lentiviral vector. Lentivirus was produced in 293T cells. In short, 293T cells were plated on a 10 cm dish the day prior to transfection. On the day of transfection, the plasmid mixture containing 9 µg TCR lentiviral plasmid, 3 µg pCMV-VSVG plasmid, 3 µg pMDLg pRRE plasmid and 3 µg pRSV-Rev plasmid were prepared with Opti-MEM (Gibco, Cat#31985070). Then, 28 µL Lipo8000 transfection reagent (Beyotime, C0533) was added into Opti-MEM and dropped into the above plasmid mixture. The complexes were incubated for 20 min at room temperature and then added to the 293T culture. Transfection media was removed 6 h later and replaced with DMEM supplemented 10% FBS. The lentivirus supernatant was harvested at 48 h and 72 h after transfection. The Universal Virus Concentration Kit (Beyotime, C2901L) was used to purify the lentivirus. TCR-deficient Jurkat 76 (J76) cells were transduced with viral supernatant. Briefly, 1 × 10^5^ J76 cells were mixed with TCR virus and polybrene (1:1000) (Beyotime, C0351) and transferred to 24-well plates. Then, the plates were centrifuged at 800g for 60 min at 30 °C. TCR expression was assessed by flow cytometry after 72h.

### *In vitro* stimulation of the J76 T cells

The MAGE-A3+HLA-A2+ PC9 and A375 cells were treated with 20 µg/mL mitomycin C for 30 min and transferred to 48/96-well plates. J76 cells expressing the exogenous TCR-1, TCR-2, TCR-12, TCR-103 and TCR-207 TCRs were co-cultured with PC9 cells at a 2:1 ratio (2×10^5^: 1×10^5^, 48-well) or A375 cells at a 2:1 ratio (0.5×10^5^: 0.25×10^5^, 96-well), respectively, and stimulated with 1 µg/mL anti-human CD28 (BioLegend, Cat#302901, US), 50 IU/mL IL-2 (SL PHARM, Recombinant Human Interleukin-2 (125 Ala) Injection). T cell activation marker CD69 (BioLegend, Cat#310909, US) and CD137 (BioLegend, Cat#309809, US) were detected at 16 h. Hoechst staining was used to detect the living PC9 or A375 cells at 96 h. The ImageJ software was used to count the living cells.

### CCK8 assay

The MAGE-A3+HLA-A2+ PC9 and A375 cells were treated with 20 µg/mL mitomycin C for 30 min and inoculated to 96-well plates (1×10^4^/well, 2.5×10^4^/well, respectively). J76 cells expressing the exogenous TCR-1, TCR-2, TCR-12, TCR-103 and TCR-207 TCRs were co-cultured with PC9 or A375 cells at a 2:1 ratio, respectively, and stimulated with 1 µg/mL anti-human CD28 (BioLegend, Cat#302901, US) and 50 IU/mL IL-2 (SL PHARM, Recombinant Human Interleukin-2 (125 Ala) Injection). After co-cultivation for 96 h, the OD values were detected by the CCK8 assays (Beyotime, Cat#C0038, China) according to the manufacturer's instructions.

### *In vivo* mouse model

In the study, 5-week-old male BLAB/c nu/nu *Mus musculus*, weighed 13 to 15 g were purchased from Guangdong Medical Laboratory Animal Center (Certificate number SCXK (Yue) 2022-0002). The study was approved overseen by the Animal Ethics and Use Committee of Jinan University. All animal procedures were approved. Nude mice were housed in vivarium, within individually ventilated cages with food and sterilized water supply. For subcutaneous injection, each nude mouse was injected with 5 × 10^6^ A375 cells in 100 µL volume PBS/matrigel (1:1 volume; Matrigel: Solarbio, #M8371).

For the treatment of cell derived xenograft (CDX) tumors, mice bearing similar sizes of tumors were randomly divided with five mice per group. 1×10^7^ TCR-T cells (TCR-2 and TCR-12) or control T cells were intravenously transplanted to each mouse for twice in the corresponding groups. IL-2 (2 × 10^5^ U each mouse) was intraperitoneally injected at the same time. Subcutaneous tumor volume was measured every 3 days with a digital caliper and calculated as the following formula: volume = (width)^2^ × length/2. One mouse in each group died occasionally.

### Cross-reactivity risk analysis of candidates TCRs

In this study, we employed a local model called TCRmodel2 to investigate the docking prediction of pMHC-TCR complexes. TCRmodel2 is an improved model based on alphafold2, specifically designed for the docking prediction of pMHC-TCR complexes 31. We performed docking predictions for the five candidate TCRs with ten peptides and HLA-A02:01 complexes. The selected ten peptides were obtained from the top-ranked HLA-A02:01-related peptides in the IEDB database, consisting of the five highest-ranked peptides from MAGE-12 and TITIN proteins. We also performed the same docking prediction for the 5 TCRs with MAGE-A3-Mp4 epitope. The docking prediction produces a stability score for reflecting the likelihood of binding between pMHC and TCR, and a higher stability score implies higher binding likelihood.

### Statistical Analysis

All statistics were performed by GraphPad prism 9, SPSS 26.0 software and R statistical package. Statistical significance was determined by t test and one-way ANOVA. *P* value less than 0.05 was considered to be statistically significant. The number of replicates, number of independent experiments performed, and *P* values for each experiment are reported in the corresponding figure legends.

## Results

### Identification of HLA-A2-restricted T cell epitopes from MAGE-A3

**Figure 1:**
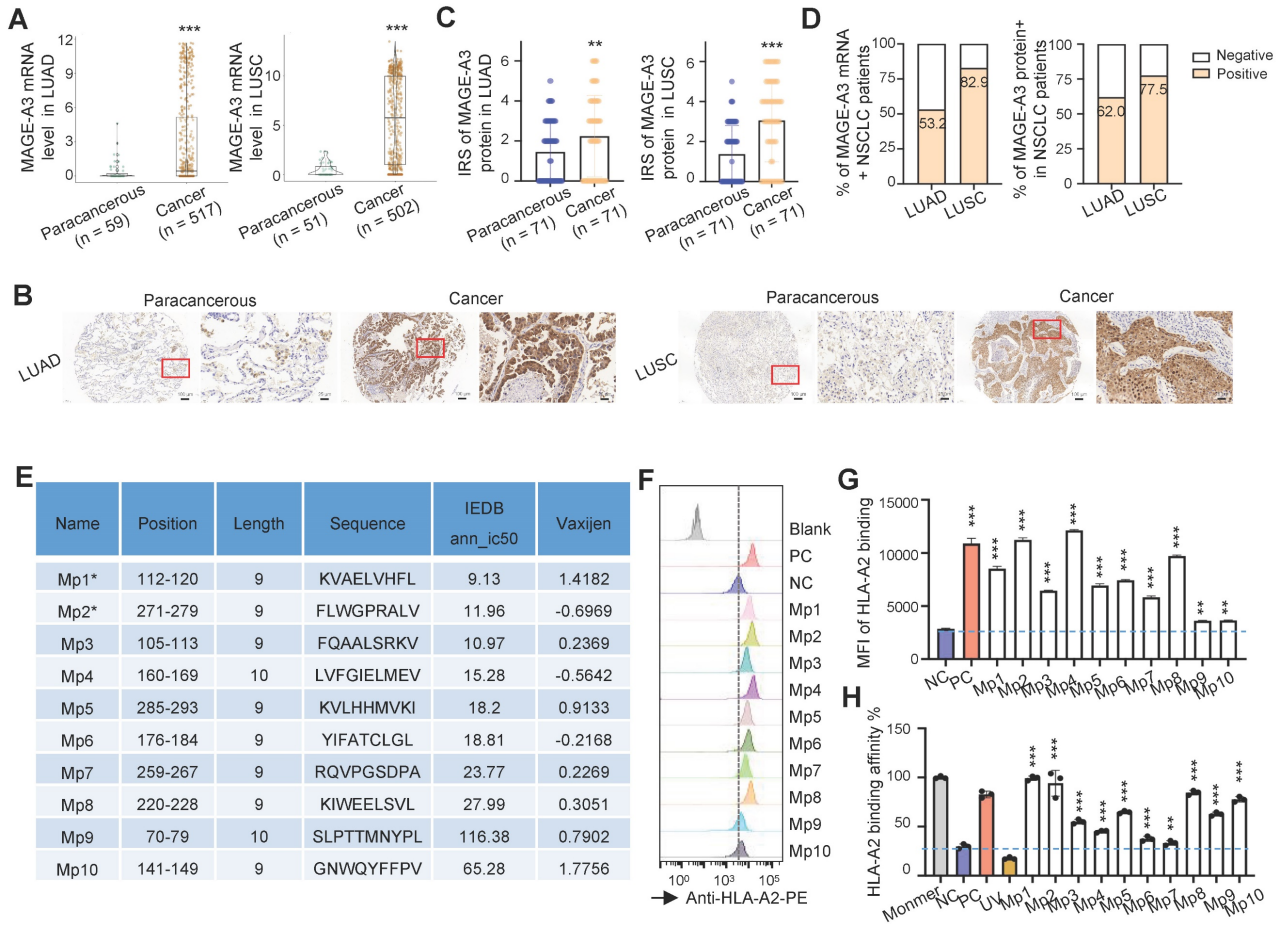
** Identification of HLA-A2-restricted T cell epitopes from MAGE-A3.** (A) Boxplots of MAGE-A3 mRNA expression level in LUAD (left, n = 59 paracancerous and n = 517 tumor) and LUSC (right, n = 51 paracancerous and n = 502 tumor) from the TCGA database. (B) Representative immunohistochemistry images of MAGE-A3 protein in LUAD (left) and LUSC (right), and the adjacent lung tissue. (C) Overall statistics of MAGE-A3 immunohistochemistry on sections from the LUAD (left) and LUSC (right) tissue microarray (n = 75). Bar charts show the medians with individual data points, **P < 0.01, ***P < 0.001. (D) Bar charts summarizing the percentage of MAGE-A3 mRNA (left) and protein (right) positive NSCLC patients, respectively. (E) Summary of the predicted HLA-A2 restricted T cell epitopes from MAGE-A3. (F-G) Comparison of the predicted MAGE-A3 epitopes binding affinity to HLA-A2 MHC-I on T2 cells. Binding capacity was presented as mean fluorescence intensity (MFI) of HLA-A2 staining, (F) is the representative plot of (G), **P < 0.01, ***P < 0.001; Blank: no peptides; NC: negative control, EBV virus peptide IVTDFSVIK; PC: positive control, flu M1 peptide GILGFVFTL. Data are shown as mean ± SD. Statistical significance was determined by one-way ANOVA. (H) Evaluation of the predicted MAGE-A3 epitopes binding to HLA-A2 by ELISA assay. Threshold for pMHC formation positivity was set as above the average OD value of the negative control. Monomer: control UV-sensitive peptide without UV irradiation. UV: control UV-sensitive peptide with UV irradiation and free from MAGE-A3 peptide exchange. Data are shown as mean ± SD. Each dot represents a single experiment. **P < 0.01; ***P < 0.001. Statistical significance was determined by one-sided t-test or one-way ANOVA.

In order to confirm MAGE-A3 is a viable target for T cells, we first checked the expression of MAGE-A3 in two subtypes of NSCLC, lung adenocarcinoma (LUAD) and lung squamous cell carcinoma (LUSC). On the mRNA level, MAGE-A3 was significantly overexpressed in LUAD (Figure 1A, left) and LUSC (Figure 1A, right) tumors than the paracancerous tissue (*P* < 0.001). Then, MAGE-A3 protein expression was evaluated by immunohistochemistry in LUAD and LUSC in two tissue microarrays, with each consisting of 75 tumor and 75 matched paracancerous slices, respectively (Table S2 and Table S3). The results confirmed that MAGE-A3 exhibited positive expression in most tumor tissue of LUAD (Figure 1B, left) and LUSC (Figure 1B, right) (Figure S1A), but not the corresponding paracancerous areas. The IRS of MAGE-A3 was significantly elevated in LUAD (Figure 1C, left) and LUSC (Figure 1C, right) compared to the paracancerous regions (*P* < 0.01). In addition, the IRS of MAGE-A3 was significantly higher in tumor stage III-IV LUAD tumors than lower grades (*P* < 0.05) (Figure S1B). Collectively, 53.2% (n = 275) and 82.9% (n = 416) of the analyzed LUAD (n = 517) and LUSC (n = 502) patients exhibited positive MAGE-A3 mRNA expression, respectively (Figure 1D, left). Furthermore, 62.0% (n = 44) and 77.5% (n = 55) of the immunohistochemistry tested LUAD (n = 71) and LUSC (n = 71) tumor samples were deemed positive in MAGE-A3 protein expression (Figure 1D, right). Overall, these results confirmed frequent high expression of MAGE-A3 in NSCLC, which can be considered as a potential immunotherapeutic target for NSCLC.

Effective antigen presentation to CD8+ cytotoxic T cells requires an antigen fragment, commonly a 9‐11 amino acid peptide sequence, to be bound to the groove of the MHC class I (MHC-I) molecules on the APCs and be effectively presented to matching TCRs. Thus, we focused on screening MAGE-A3 antigenic epitopes that could bind to MHC-I and in turn induce CD8+ T cell immune response. To do so, we first predicted the potential HLA-A2 restricted epitopes with IEDB. We focused on HLA-A2 MHC-I molecule as it is the most common HLA subtype among Chinese population 22, 23. In total, 10 putative MAGE-A3 epitopes (named as Mp1 to Mp10) were predicted, of which Mp1 and Mp2 were also previously reported by Chinnasamy et al. as MAGE-A3 epitopes 32. The peptides of the predicted epitopes were then synthesized for MHC-I binding and peptides exchange efficiency screening using T2 cells 25 (Figure 1E). The T2 binding assay indicated that all these 10 MAGE-A3 epitopes exhibited significant MHC-I binding capability compared to the negative control (NC) (*P* < 0.01) (Figure 1F-G). In addition, all of these epitopes could form peptide-MHC monomers and further tetramers (*P* < 0.001) (Figure 1H).

### T cell stimulation with predicted MAGE-A3 epitopes

**Figure 2:**
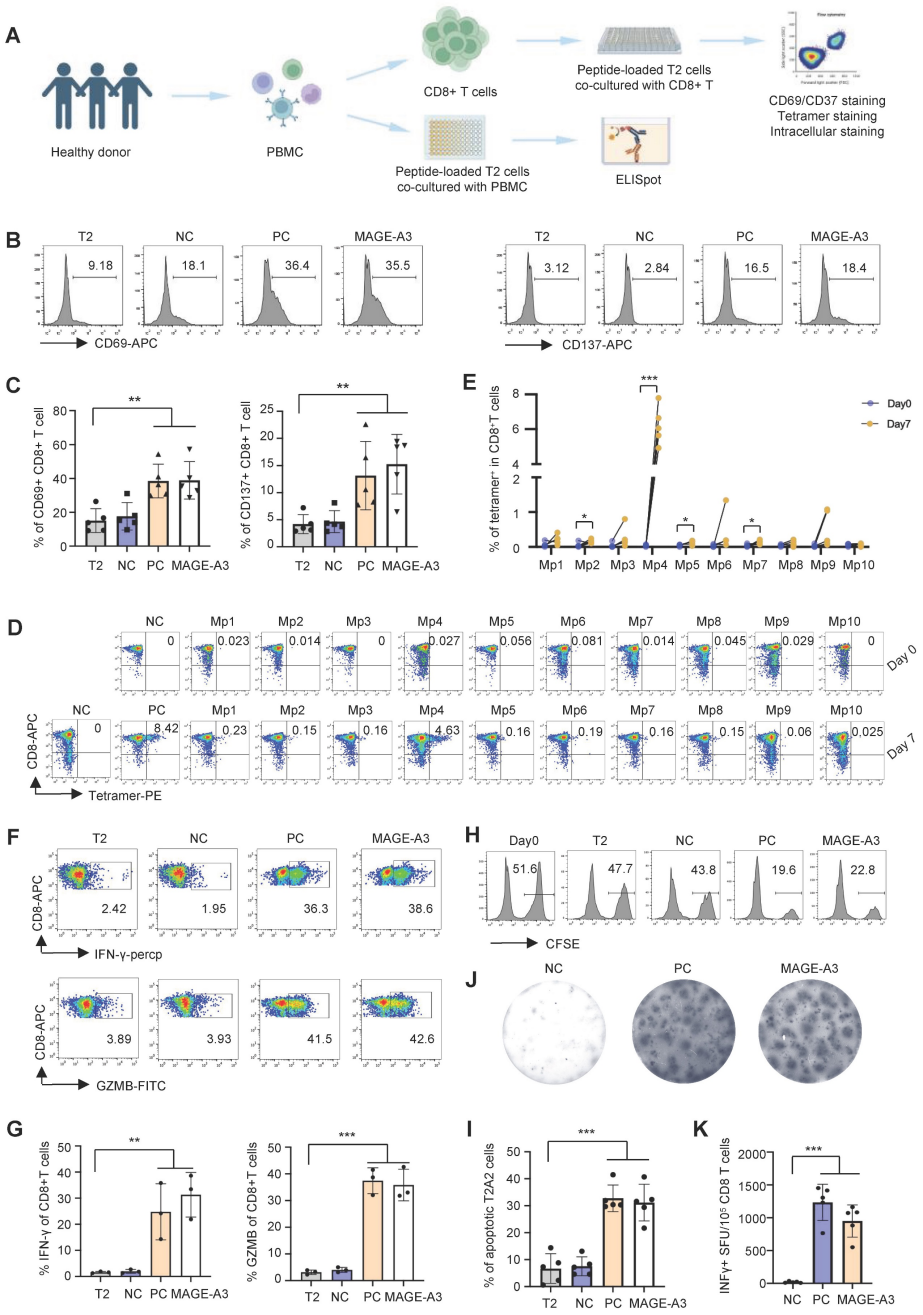
** T cell activation and cytotoxicity induced by epitopes from MAGE-A3.** (A) Schematics showing the *in vitro* screening of MAGE-A3 epitope-specific T cells by artificial APCs. CD8+ T cells from HLA-A2+ healthy donors were co-cultivated with peptide-loaded T2 cells. The T cell activation marker CD69 and CD137 were analyzed at 16h. Tetramer+ CD8+ T cell were detected at day 0 and day 7. Intracellular staining (IFN-γ and GZMB) was used to detect T cell cytotoxicity at day 7. (B-C) Representative FACS results (B) and overall summary statistics (C) of CD8+ T cell activation marker CD69 and CD137 expression after co-cultivation with T2 cells loaded with MAGE-A3 peptides (n = 5). T2: CD8+ T cells co-cultivated with PBS treated T2 cells; NC: CD8+ T cells co-cultivated with EBV peptide loaded T2 cells; PC: CD8+ T cells co-cultivated with flu peptide loaded T2 cells. CD69 and CD137 expression was detected by FACS 16 hours post-cocultivation. (D) Representative FACS plots showing the stimulation of the CD8+ T cells by tetramers. Top row, day 0; bottom row, day 7. CD8+ T cells from healthy donors were co-cultivated with T2 cells loaded with MAGE-A3 peptides for activation. (E) Summary statistics of epitope specific CD8+ T cell (n = 5) before (day 0) and after 7 days stimulation by distinct MAGE-A3 epitopes. (F) Representative FACS plots of IFN-γ (top) and GZMB (bottom) in CD8+ T cells after epitope stimulation for 7 days. Values in each panel indicate the percentage of IFN-γ+CD8+ or GZMB+CD8+ T cells, respectively. (G) Corresponding summary statistics of IFN-γ (left) and GZMB (right) positive CD8+ T cells (n = 3). (H-I) Epitope specific CD8+ T cell mediated cytotoxicity evaluation after 7 days of cell culturing. Representative FACS plots result (H) and corresponding summary statistics (I) of apoptotic cells. CFSE+ T2 cells were counted as survived target cells and the percentage of apoptotic cells was calculated by 50% minus the percentage of survived cells (n = 5), statistical significance was determined by kruskal wallis H test. (J-K) Exemplary microscopy image (J) and summary statistics (K) for anti-IFN-γ ELISpot assay on PBMC cells from HLA-A2+ healthy donors stimulated by distinct epitopes (n = 5). NC: PBMC co-cultivated with EBV peptide loaded T2 cells; PC: PBMC co-cultivated with T2 cells in the existence of anti-CD3 mAb CD3-2; MAGE-A3: PBMC co-cultivated with MAGE-A3 peptides loaded T2 cells. Data are shown as mean ± SD. **P < 0.01; ***P < 0.001. Each dot represents a single experiment. Statistical significance was determined by one-sided t-test or one-way ANOVA.

We then established an *in vitro* artificial APC system to screen T cell epitopes from MAGE-A3 using CD8+ T cells from healthy HLA-A2+ donors (Figure 2A). MAGE-A3-Mp1 to MAGE-A3-Mp10 peptide-loaded T2 cells were co-cultured with CD8+ T cells from healthy HLA-A2+ donors, and subsequently the immune responses from the CD8+ T cells were evaluated. The results showed that the CD8+ T cells exhibited significantly increased activation upon stimulation by the MAGE-A3 epitopes, as indicated by the CD69 and CD137 FACS signals, comparing to the controls (Figure 2B-C) (*P* < 0.01 and *P* < 0.01, respectively). Next, tetramer staining was used to detect MAGE-A3 epitope-specific CD8+ T cells before and after stimulation. The results showed MAGE-A3-Mp4 epitope-specific CD8+ T cells had the most significant increment after 7 days co-culturing (*P* < 0.001) (Figure 2D-E, Figure S2A). This epitope was also previously reported by another MAGE-A3 related cancer vaccine study 33 In addition, the proportion of MAGE-A3-Mp1 and MAGE-A3-Mp2 epitope-specific CD8+ T cells increased by 237% and 199% after stimulation, respectively (Figure 2D-E), which is consistent with the previous study concluding Mp1 and Mp2 were immunogenic epitopes 32.

Next, we measured IFN-γ and GZMB production by intracellular staining to evaluate the cytotoxicity of the CD8+ T cells after MAGE-A3 epitopes stimulation. The results showed that both IFN-γ and GZMB production levels increased significantly from the MAGE-A3 epitopes treated group comparing to the negative control (Figure 2F-G, Figure S2A) (*P* < 0.01 and *P* < 0.001, respectively). The cytotoxicity assay also showed enhanced target cell killing effect of CD8+ T cells after stimulation by MAGE-A3 epitopes, indicated by the significantly increased proportion of apoptotic T2 cells (*P* < 0.001) (Figure 2H-I, Figure S2B). In addition, ELISpot experiment also confirmed significantly higher IFN-γ production from healthy donors after stimulation by MAGE-A3 epitopes (*P* < 0.001) (Figure 2J-K). Taken together, the identified MAGE-A3 epitopes showed strong antigenicity, and in particular, CD8+ T cells from HLA-A2+ donors exhibited the strongest specificity to MAGE-A3-Mp4.

### Detection of primary MAGE-A3-specific CD8+ T cells from NSCLC patients

**Figure 3:**
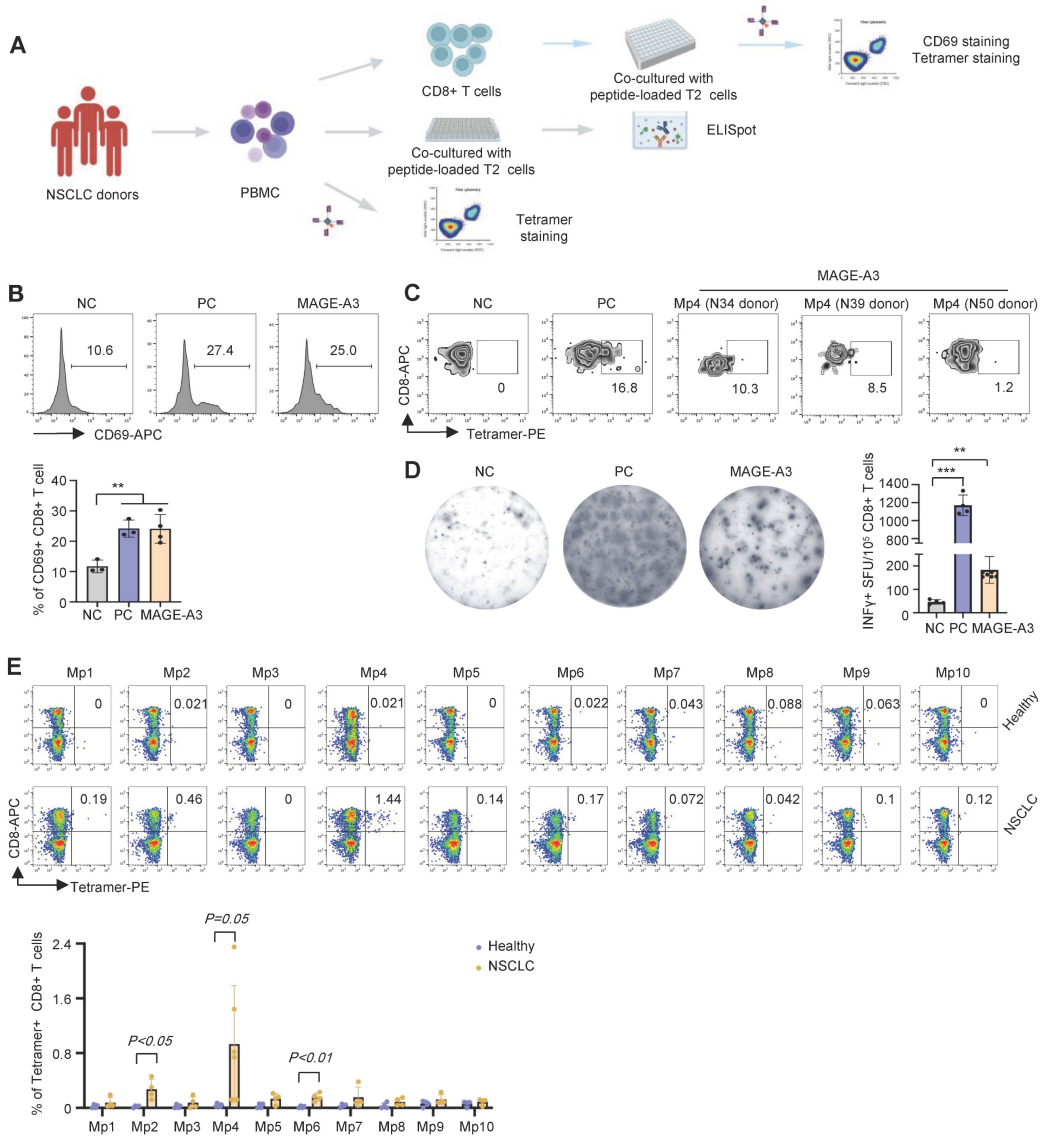
** Characterization of cytotoxic effects of MAGE-A3 epitope-specific T cells.** (A) Schematics showing the procedure used to verify immunogenic MAGE-A3 epitopes in HLA-A2+ NSCLC patients. (B) Representative FACS plots (up) and overall summary (down) of CD8+ T cell activation marker CD69 expression after co-cultivation with T2 cells loaded with MAGE-A3 peptides (n = 3). (C) Representative FACS plots showing the stimulation of the CD8+ T cells by tetramer prepared with MAGE-A3-Mp4 epitope (n = 3). CD8+ T cells from NSCLC donors were co-cultivated with T2 cells loaded with MAGE-A3 peptides for 3 days. (D) Representative images (left) and summary statistics (right) for anti-IFN-γ ELISpot assay (n = 7 for mixed; n = 4 for NC and PC). PBMC cells from HLA-A2+ NSCLC donors were co-cultivated with T2 cells loaded with MAGE-A3 peptides for 48 hours. (E) Up: Representative FACS plots of T cell staining with the indicated tetramers from the PBMC of HLA-A2+ healthy donors or HLA-A2+ NSCLC patients. Down: Percentage comparison of tetramer+CD8+ T cells between healthy donors (n = 4) and NSCLC patients (n = 6 for Mp4; n = 4 for the others). Data are shown as mean ± SD. **P < 0.01; ***P < 0.001. Each dot represents a single experiment. Statistical significance was determined by one-sided t-test or one-way ANOVA.

To further verify the antigenicity of the candidate MAGE-A3 epitopes, a cohort including 25 HLA-A2+ NSCLC donors was recruited (Table S1) for epitope-specific CD8+ T cells screening and characterization (Figure 3A). Total CD8+ T cells from the NSCLC donors were sorted and co-cultured with T2 cells loaded with mixed MAGE-A3 epitopes. Upon stimulation by the MAGE-A3 epitopes, the primary CD8+ T cells from these donors exhibited significantly increased activation indicated by CD69 FACS signals, comparing to the NC (*P* < 0.01) (Figure 3B). In addition, based on the MAGE-A3-Mp4 tetramer staining after co-cultivation for 3 days, we could detect considerable amount of MAGE-A3-Mp4 epitope-specific CD8+ T cells in the HLA-A2+ donors (Figure 3C). Furthermore, ELISpot assay showed significantly increased IFN-γ production from the NSCLC donors after stimulation (Figure 3D) (*P* < 0.01). Next, we used MAGE-A3-Mp1 to MAGE-A3-Mp10 epitope tetramers to compare specific CD8+ T cells from healthy and NSCLC donors. The results showed that NSCLC donors had increased amount of MAGE-A3-Mp2 (*P* < 0.05), MAGE-A3-Mp4 (*P* = 0.05) and MAGE-A3-Mp6 (*P* < 0.01) tetramer+CD8+ T cells than the healthy controls (Figure 3E). These results suggested that the candidate MAGE-A3 epitopes could stimulate to produce functional antigen-specific CD8+ T cells in the NSCLC patients, and in particular, the MAGE-A3-Mp2, MAGE-A3-Mp4 and MAGE-A3-Mp6 epitope-specific CD8+ T cells were likely already present in the patients.

### Characterization of MAGE-A3 epitope-specific T cells by single-cell sequencing

Single-cell sequencing is a powerful tool for identifying T cells with TCRs targeting tumor antigens and can provide complete pairing information for the α and β chains of TCRs 34, 35. Single-cell sequencing can also be used to distinguish potential cell clusters with different functions, which is important for accurate screening TCRs with killing function.

**Figure 4:**
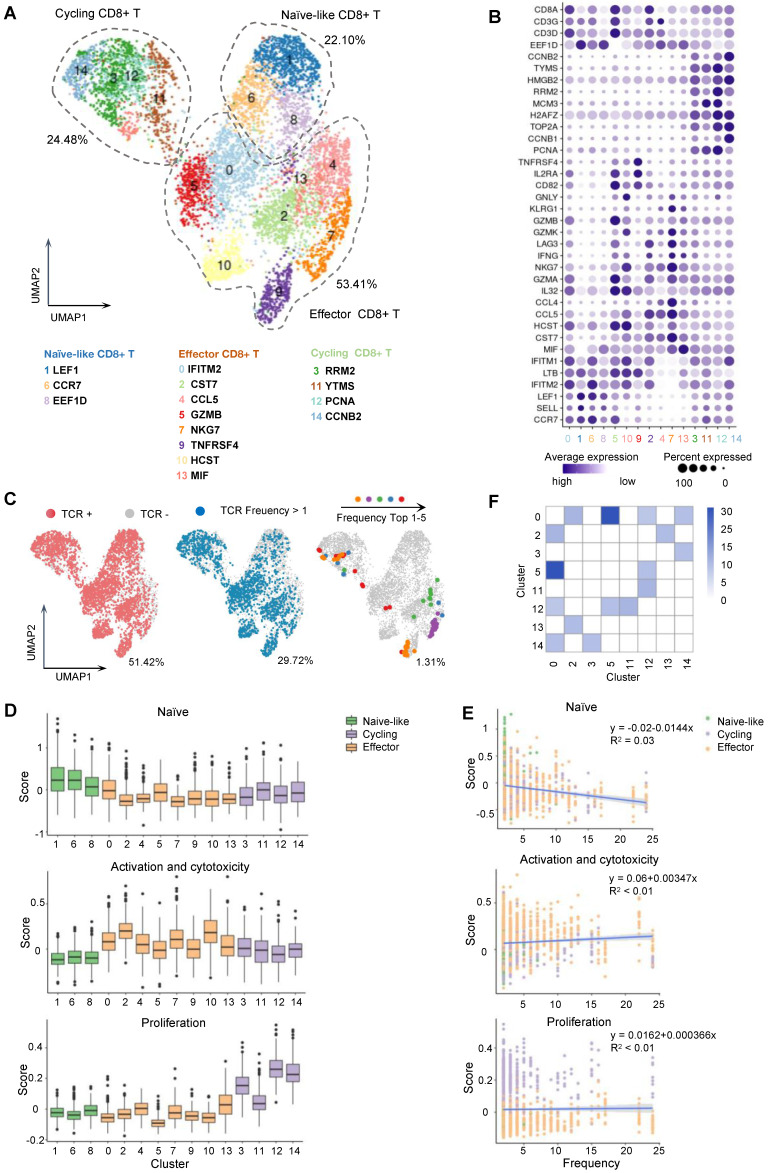
** Single-cell transcriptome and TCR landscape of CD8+ T cells specific to MAGE-A3-Mp4 epitopes.** (A) Uniform manifold approximation and projection (UMAP) visualization of the scRNA-seq data from 5891 tetramer sorted MAGE-A3-Mp4 epitope-specific CD8+ T cells. The identified cell clusters (n = 15) are depicted with distinct colors. Cluster-specific genes are shown adjacent to each cluster ID, respectively. (B) Dot plot of marker genes for each T cell subtypes. Color-scale shows the average normalized expression of marker genes in each subtype, and dot size indicates the percentage of cells within each cell cluster expressing the marker gene. The cluster IDs on x-axis is the same as (A). (C) UMAP visualization with total TCR sequence detection information (left), the TCR clonotype expansion (clonotype frequency > 1) information (middle) and the top 5 most frequent TCR clonotype information for MAGE-A3-Mp4 epitope. (D) Single-cell transcriptome-derived CD8+ T cell naivety, proliferation and activation, and cytotoxicity score comparison between cell clusters. Gene panels used for naivety, activation and cytotoxicity, and proliferation score calculation are listed in Table S5. (E) Scatter-plot visualization of CD8+ T cell TCR clonotype frequency (x-axis) vs. cell naivety, activation and cytotoxicity, and proliferation score, respectively. Each dot represents a CD8+ T cell, with color corresponds to its annotated subtype. Blue lines are fitted by linear model with grey area indicating the 95% confidence band. Spearman correlations (ρ) are also shown. (F) Heatmap visualization of numbers of TCR clonotypes shared by two CD8+ T cell clusters. Only cell clusters with shared clonotypes > 10 with at least one other cluster are shown. For boxplots, the outlines of the boxes represent the first and third quartiles. The line inside each box represents the median, and boundaries of the whiskers are found within the 1.5×IQR value.

Based on the CD8+ T cell stimulation and tetramer staining results, MAGE-A3-Mp4 epitope was finally selected as a major T cell epitope from MAGE-A3. To further characterize MAGE-A3 antigen-specific CD8+ T cells, CD8+ T cells from two healthy HLA-A2 donors were co-cultivated with peptide-loaded T2 cells as was described in Figure 2A. The MAGE-A3-Mp4 epitope-specific CD8+ T cells were sorted after tetramer staining (Figure S3A-C) and used for paralleled single-cell RNA-seq and TCR V(D)J sequencing. Healthy donors were used here for a more comprehensive and unbiased profiling of CD8+ T cells after encountering the MAGE-A3 antigens 28. In total, we produced high-quality scRNA-seq and scTCR-seq data from 5,891 CD8+ T cells with paired TCR chains potentially specific to MAGE-A3-Mp4 epitope. The CD8+ T cell were divided into 15 clusters based on the transcriptome profiles and the specific marker genes for each cluster were identified (Figure 4A-B, Figure S4A-C; Table S4). Cluster 1, 6 and 8 were annotated as naïve-like CD8+ T cells characterized by commonly high expression of CCR7, SELL and LEF1 36. Cluster 0, 2, 4, 5, 7, 9 ,10 and 13 were annotated as effector CD8+ T cells according to the reported markers (e.g., IFNG, GZMB, NKG7, CST7 and GNLY) 37-39. Lastly, cluster 3, 11, 12 and 14 featured with high expression of RRM2, YTMS, PCNA and CCNB2, which are related to cell proliferation and cell cycle 28, were named as cycling CD8+ T cells (Figure 4A-B). Re-analysis of the scRNA-seq of two HLA-A2 healthy young adults from our previous study 40 showed that the CD8+ T cells without stimulation mainly included naïve, effector, memory and other CD8+ T cells (Figure S5A-B), of which naïve CD8+ T cells accounted for majority (50.9%) (Figure S5C). However, the naïve-like CD8+ T cells only accounted for 22.10% in MAGE-A3 peptides stimulated CD8+ T cells (Figure 4A), which is related to asymmetric division of naïve CD8+ T cells upon antigen stimulation 41.

The CD8+ T cells with definitive paired TCR chains accounted for 51.42% of all analyzed cells (Figure 4C; Figure S5D-E). Further, the analyzed cells having TCRs with clonotype expansion (clonotype frequency > 1) accounted for 29.72%, and the ones with top 5 clonotypes collectively accounted for 1.31%, which were mostly detected in CD8+ T cells in effector and proliferating state (Figure 4A, C; Figure S5D-E).

Next, we sought to functionally characterize the MAGE-A3-Mp4 epitope-specific CD8+ T cells with gene panels related to T cell naivety, proliferation and cytotoxicity (Table S5). The results suggested that naïve-like CD8+ T cell clusters were relatively more naïve, whereas effector CD8+ T cell clusters were relatively more activated with higher cytotoxicity, and cycling CD8+ T cells were indeed more proliferative (Figure 4D). Furthermore, the CD8+ T cells with higher TCR clonotype expansion tended to have lower cell naivety but higher cytotoxicity and are weakly more proliferative (Figure 4E), consistent with the fact that effector CD8+ T cells with higher cytotoxicity tended to be more clonally expanded 42. Lastly, the high frequency of TCR clonotypes shared between cluster 2, 5, 12, 14 and 0 potentially implied the transformation of naïve CD8+ T cells to cycling and effector CD8+ T cells upon stimulation by MAGE-A3-Mp4 (Figure 4F). Overall, these single-cell transcriptome and TCR clonotype expansion characterization results suggested the analyzed CD8+ T cells were indeed specific to MAGE-A3-Mp4 epitope, and their TCR peptide sequences can be potentially utilized for NSCLC TCR-T therapy.

### Identification of MAGE-A3 specific TCRs by machine-learning

**Figure 5:**
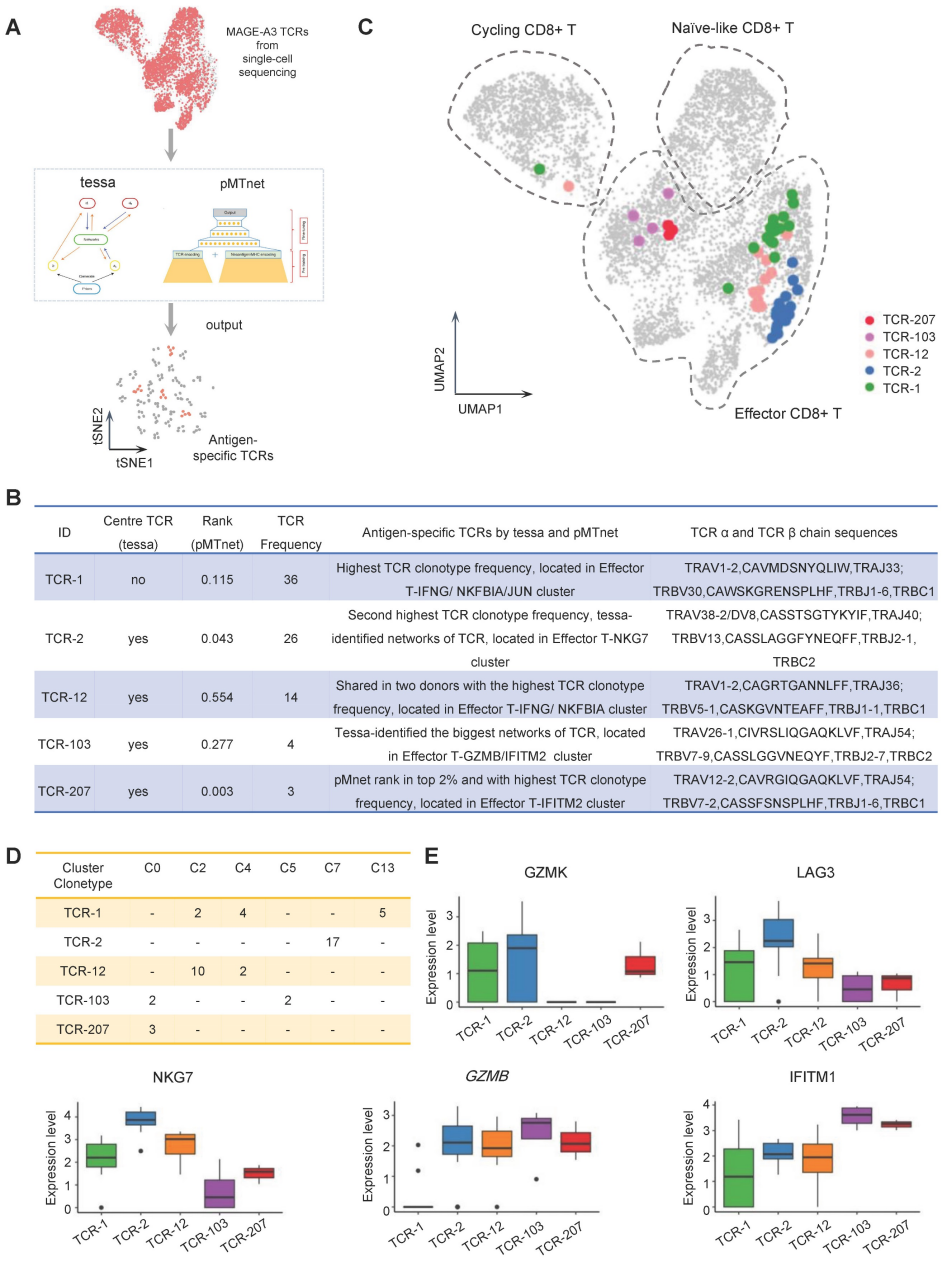
** Antigen-specific TCRs screening by machine-learning.** (A) Schematic workflow for inferring MAGE-A3 antigen specificity for the CD8+ T cells from health donors using the tessa and pMTnet machine-learning framework, respectively. (B) The detailed information of the selected candidate TCRs. (C) UMAP visualization of the MAGE-A3-Mp4 epitope-specific CD8+ T cells with the 5 selected candidate TCR sequences information projection. (D) The clonotype expansion distribution information of the 5 selected candidate TCRs in the corresponding CD8+ T cell cluster. (E) Boxplot of selected gene expression level of CD8+ T cells with the 5 selected candidate TCRs. For boxplots, the outlines of the boxes represent the first and third quartiles. The line inside each box represents the median, and boundaries of the whiskers are found within the 1.5×IQR value.

TCR-T immunotherapy targeting the immunogenic tumor antigen can effectively clear the tumor without obvious side effects. However, most endogenous tumor-specific TCRs have low affinity for tumor-associated pMHCs that only weakly activate the TCR-T cells they bind to 8, 43. Therefore, screening for TCRs specific to tumor antigens with suitable affinity is very challenging. Tessa (TCR functional landscape estimation supervised with scRNA-seq analysis) is a Bayesian model to screen the T cell cloning types with the strongest immune response from sc-RNAseq and sc-TCRseq data 29. pMTnet (pMHC-TCR binding prediction network), a transfer learning-based model, was previously used to predict TCR binding specificities of the neoantigens and T cell antigens presented by MHC-I 30. To identify the suitable candidate TCR clonotypes for NSCLC TCR-T therapy, we applied the tessa and pMTnet machine-learning algorithms on the single-cell sequencing data from the CD8+ T cells stained with MAGE-A3-Mp4 tetramers (Figure 5A). By overall consideration of the TCR clonotype expansion and results from tessa and pMTnet, eventually 5 TCR clonotypes (namely TCR-1, TCR-2, TCR-12, TCR-103 and TCR-207) were selected for downstream analysis and validation (Figure 5B-C).

Specifically, TCR-1 had the highest clonotype frequency from the single-cell sequencing data. TCR-2 had the second highest TCR clonotype frequency and was a center TCR of a TCR network with high specificity to MAGE-A3-Mp4 according to the tessa analysis. Additionally, the pMTnet analysis predicted that TCR-2 was of high affinity to MAGE-A3-Mp4 HLA-A2 pMHC (pMTnet rank value = 0.043). TCR-12 was shared between the two CD8+ T cell donors with the highest TCR clonotype frequency. TCR-103 was the center TCR of the biggest TCR network identified by tessa. TCR-207 had the highest TCR clonotype frequency among the top 2% TCRs ranked by pMTnet. Coincidentally, these 5 selected TCRs were mapped to CD8+ T cells mainly in effector and proliferating state (Figure 5C) and most were shared between multiple cell clusters (Figure 5D). Furthermore, we found that the effector CD8+ T cell marker genes such as GZMB, NKG7 and IFITM1 were highly expressed in cells with TCR-2, TCR-12 and TCR-207 clonotype, while the exhaustion CD8+ T cell marker genes such as GZMK and LAG3 were highly expressed in cells with TCR-2 (Figure 5E). Overall, these findings implied CD8+ T cells with these 5 selected TCR clonotypes could potentially have high tumor killing effect by targeting the MAGE-A3-Mp4 epitope.

### *In vitro* and *in vivo* anti-tumor assessment for candidate TCRs

**Figure 6:**
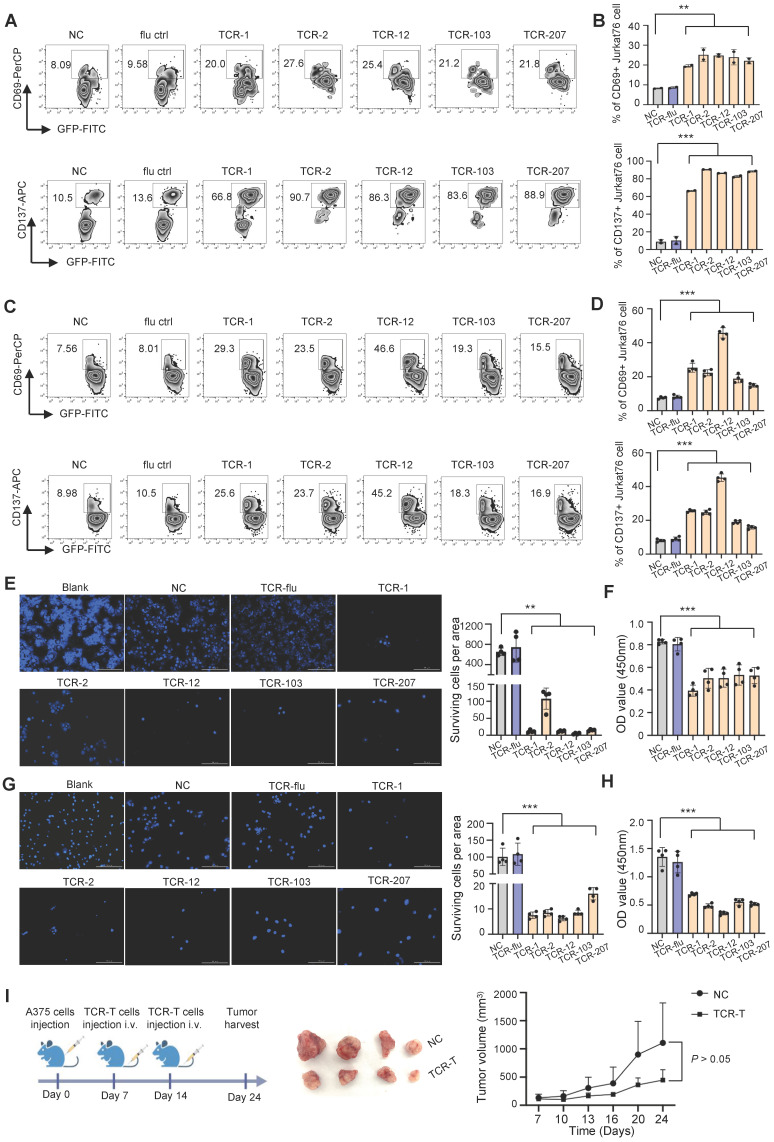
** T cells expressing the candidate TCRs responds to MAGE-A3 antigen *in vitro and in vivo.*** (A-B) Representative FACS plots (A) and overall summary statistics (B) of J76 cells expressing activation marker CD69 and CD137. J76 cells expressing TCR-1, TCR-2, TCR-12, TCR-103 and TCR-207 were co-cultured with PC9 lung cancer cells. CD69 and CD137 expression was detected by FACS 16 hours post-cocultivation (n = 2). (C-D) Same as (A-B), but for J76 cells expressing TCR-1, TCR-2, TCR-12, TCR-103 and TCR-207 co-cultured with A375 melanoma cells. CD69 and CD137 expression was detected by FACS 16 hours post-cocultivation (n = 4). (E) Representative Hoechst staining of PC9 cells (left) and summary statistics of surviving cell counts (right; n = 4). (F) Bar plots showing the summary statistics of OD value (n = 4). The PC9 cells were co-cultivated with J76 cells expressing the candidate TCRs for 96h. (G) Representative Hoechst staining of A375 cells (left) and summary statistics of surviving cell counts (right; n = 4). (H) Bar plots showing the summary statistics of OD value (n = 4). The A375 cells were co-cultivated with J76 cells expressing the candidate TCRs for 96h. (I) Experiment overview of *in vivo* anti-tumor assessment (left), visual observation of the tumors (middle) and tumor growth curves of mice grouped by treatment with TCR-T (TCR-2 and TCR-12) cells (1×10^7^) or control-T cells (1×10^7^) (right), mice (n=4). Data are shown as mean ± SD. **P < 0.01; ***P < 0.001. Each dot represents a single experiment. Statistical significance was determined by one-sided t-test or one-way ANOVA.

To examine if the candidate TCRs can recognize MAGE-A3 antigen for T cell activation, we performed *in vitro* tumor antigen stimulation experiment with T cells artificially expressing these TCRs. Firstly, J76 cells (a human T cell line with devoid TCR α and β chains) was used to artificially express the 5 candidate TCRs, respectively, and the expression of TCR was verified by flow cytometry (Figure S6A). Secondly, for the target tumor cells, we verified that the lung cancer cell line PC9 and melanoma cell line A375 is HLA-A2 positive and expresses MAGE-A3 protein (Figure S6B-C). Lastly, the J76 cells expressing the 5 candidate TCRs were co-cultured with PC9 or A375 cells, respectively, for T cell activation assessment. After 16 hours cultivation, the J76 cells with the candidate TCRs had significantly higher expression of CD69 and CD137 (*P* < 0.01, *P* < 0.001, respectively) (Figure 6A-D).

Next, we examined cell-mediated cytotoxicity by co-culturing PC9 cells or A375 cells with the J76 cells expressing TCR-1, TCR-2, TCR-12, TCR-103 and TCR-207, respectively. Compared to the NC group, the living PC9 and A375 cells co-cultured with J76 expressing the candidate TCRs all decreased significantly (*P* < 0.01 and *P* < 0.001, respectively) (Figure **6**E-H). Meanwhile, there is no difference in the living PC9 and A375 cells when co-cultured with J76 expressing flu virus specific TCR, indicating no recognition of MAGE-A3 antigen by non-specific TCR. In summary, J76 cells with artificial expression of the candidate TCRs could effectively recognized MAGE-A3 tumor antigens presented on HLA-A2 pMHC and subsequently lead to target cell lysis.

In addition, we tested whether TCR-T cells engineered with the candidate TCRs affects *in vivo* tumor growth. TCR-2-T and TCR-12-T cells were evaluated in CDX models established with HLA-A2+MAGE-A3+ A375 cells. The mice bearing tumors were injected with 1×10^7^ TCR-T cells (TCR-2 and TCR-12, mixed) intravenously on day 7 and day 14 (Figure 6I). Non-transduced T cells were used as control. It was shown that the tumors volume decreased after the treatment of TCR-T cells expressing TCR-2 and TCR-12 (Figure 6I). The result demonstrated that TCR-T cells engineered with TCR-2 and TCR-12 produced tumor-suppressive effects *in vivo*.

### Cross-reactivity risk analysis of candidates TCRs

**Figure 7:**
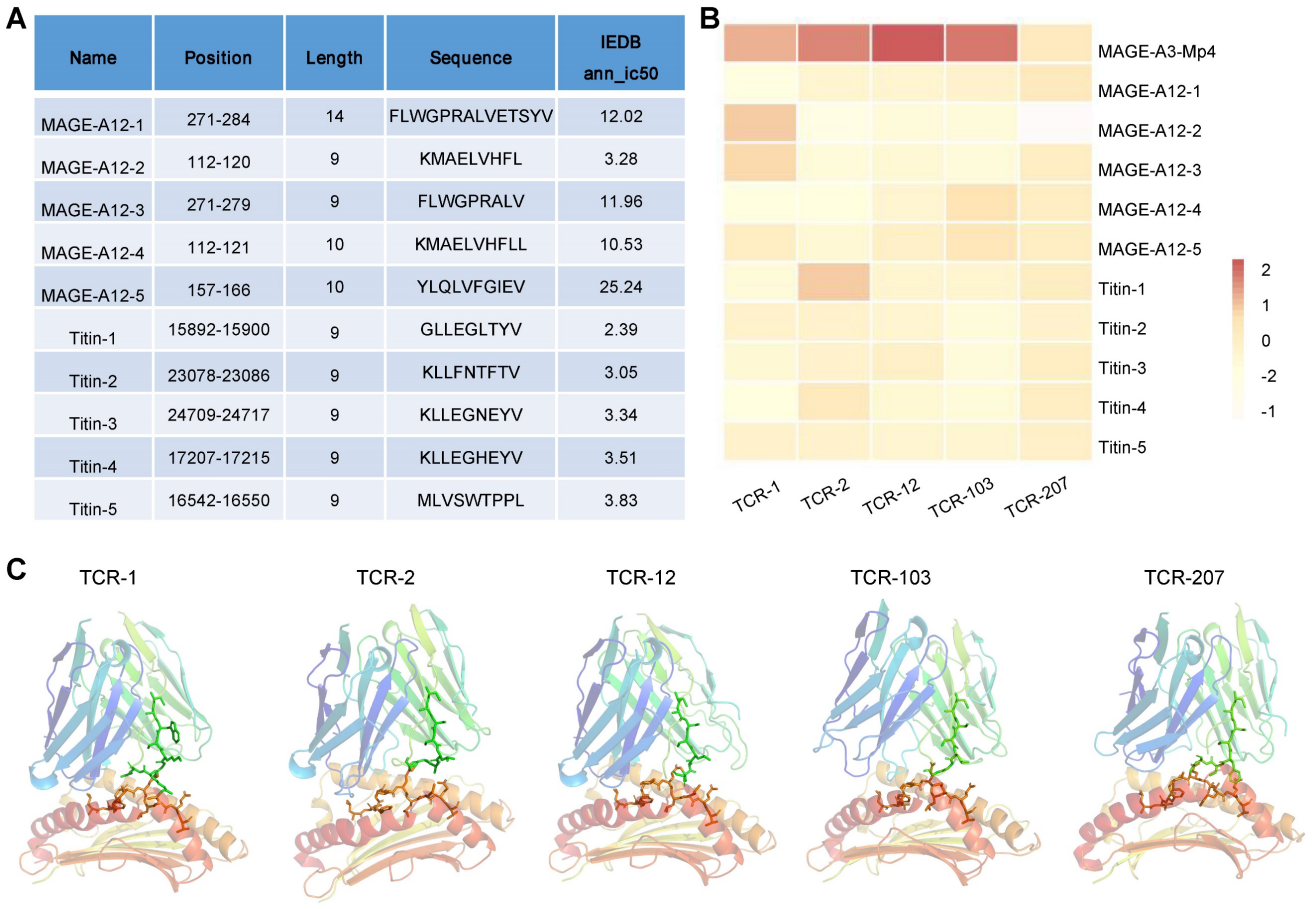
** Evaluation of cross-reaction with peptides from MAGE-A12 and Titin by candidate TCRs.** (A) Summary of the predicted top 5 HLA-A2 restricted T cell epitopes from MEGA-12 and Titin protein. (B) Heatmap visualization of the docking prediction results between the 5 candidate TCRs and HLA-A2 presenting distinct epitopes. Color intensity represents different docking stability score, with darker colors indicating higher score and suggesting more stable pMHC-TCR complex. (C) The simulated pMHC-TCR complex docking structure of the 5 candidate TCR with MAGE-A3-Mp4: the top part is the structure of TCR and the bottom is the structure of HLA-A2 presenting the MAGE-A3-Mp4 peptide.

MAGE-A3-specific TCR-T was previously reported to cross-react with a peptide from a muscle protein Titin 21, and epitope of MAGE-A12 expressed in human brain 19, which lead to off-target toxicity. Here, we aim to evaluate whether the candidate TCRs identified in this study would also recognize peptides from Titin and MAGE-A12 and cause cross-reactivity. To do so, we employed a local protein docking prediction model called TCRmodel2 31 to investigate the binding between pMHC and TCR, which could help to evaluate the cross-reactivity risk of candidate TCRs. We first identified the top 5 HLA-A02:01 restricted epitope peptides from MAGE-A12 and Titin protein, respectively, from the IEDB database (Figure 7A). These 10 epitopes presented by HLA-A2 were tested for binding stability with our 5 candidate TCRs. In addition, MAGE-A3-Mp4 epitope was also tested. To provide an intuitive comparison of our prediction results, we visualized the TCRmodel2 predictions of TCR-pMHC binding stability using heatmap. The result showed that MAGE-A3-Mp4-TCR12 had the best binding stability, and the 5 candidate TCRs had low binding stability with epitopes from MAGE-A12 and TITIN, thus low risk of cross-reaction (Figure 7B-C).

### ICB prognosis prediction by MAGE-A3-specific TCRs

**Figure 8:**
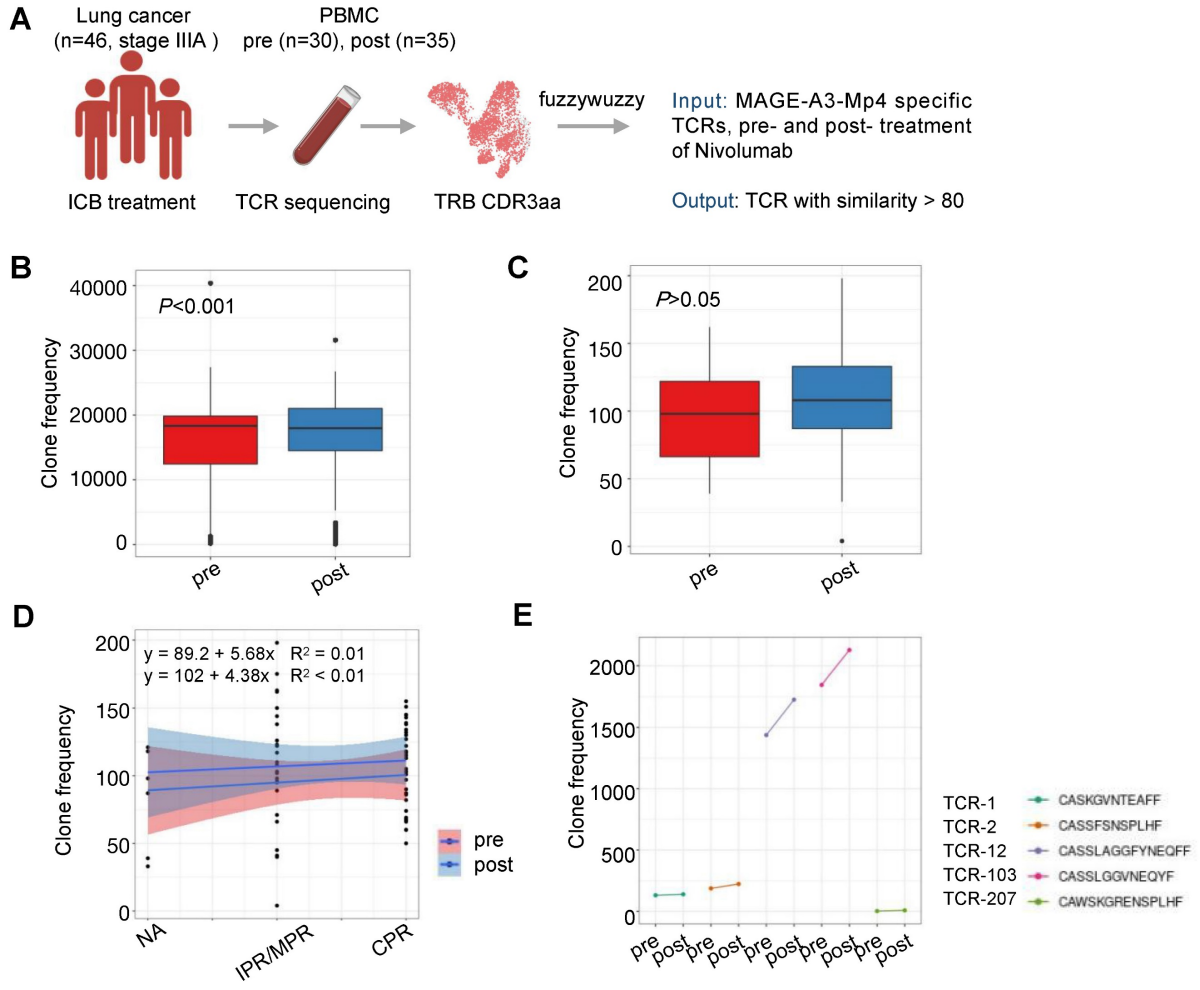
** Characterization of MAGE-A3-specific TCR clonotype expansion in lung cancer patients responding to ICB treatment.** (A) Schematics of analysis workflow (also see Methods). (B) Boxplot comparison of the number of TCRs from lung cancer patients before and after ICB treatment matching MAGE-A3-Mp4 specific TCRβ CDR3 sequences from our single-cell sequencing data. 80% CDR3 amino acid sequence similarity was considered as a match. For boxplots, the outlines of the boxes represent the first and third quartiles. The line inside each box represents the median, and boundaries of the whiskers are found within the 1.5IQR value. Statistical significance was determined by Wilcoxon. (C) Same as B, but for comparing the TCR repertoires of lung cancer patients pre- and post-treatment to the 5 selected candidate TCR sequences (i.e., TCR-1, TCR-2, TCR-12, TCR-103 and TCR-207). Statistical significance was determined by paired t test. (D) Scatter plot showing the number of TCRs from lung cancer patients pre- and post-treatment in different pathologic response condition matching the 5 candidate TCRs. IPR, minor pathologic response. MPR, major pathologic response. NA, not otherwise specified. CPR, complete pathologic response. (E) Statistics of sum of TCRs from lung cancer patients pre- and post-treatment matching each of the 5 candidate MAGE-A3-Mp4 specific TCRs.

The therapeutic efficacy of ICB depends on the antigen-specific cell mediated tumor destruction 44. Specifically, the ICB response depends primarily on the presence of highly polymorphic TCRs in the individual. Previously, studies have reported that the TCR repertoire diversity were associated with response to immunotherapy in multiple cancers 45-47. However, the predictive value and response of MAGE-A3 antigen-specific T cell clones to ICB immunotherapy in patients with lung cancer is unclear. To understand if MAGE-A3 antigen-specific TCRs discovered in our study is predictive for ICB immunotherapy outcome, we compared the TCR repertoires of 46 lung cancer treated with three rounds of nivolumab 48 with the TCR clonotypes derived from the MAGE-A3-Mp4 antigen-specific CD8+ T cell scTCR-seq data, and in particular, the 5 candidate TCRs deemed suitable for TCR-T cell design (Figure 8A). We found that after ICB therapy, the patients had significantly more TCRs matching the MAGE-A3-Mp4 antigen-specific TCR clonotypes (*P* < 0.001) (Figure 8B). If only the 5 candidate TCR clonotypes were used for comparison, the matching MAGE-A3-Mp4 antigen-specific TCR clonotypes also increased after ICB treatment (*P* > 0.05) (Figure 8C). Furthermore, patients with complete pathologic response (CRP) tended to have larger number of TCRs matching the 5 candidate TCR clonotypes than the ones only had minor or major pathologic response (IPR/MPR) (Figure 8D). Lastly, TCR-12 and TCR-103 exhibited higher number of matching TCRs from the patients than the other 3 candidate TCRs, and the number of matching TCRs greatly increased after ICB therapy (Figure 8E). Collectively, these results indirectly proved that the candidate TCRs from our study were indeed specific to lung cancer tumor antigens.

## Discussion

In this study, using *in vitro* artificial APC system, scRNA-seq, scTCR-seq and machine-learning, we comprehensively identified MAGE-A3 tumor epitopes and corresponding TCRs in human lung cancer. We identified a MAGE-A3-derived epitope Mp4 (LVFGIELMEV) with high antitumoral immune response, and was properly processed and presented on human HLA-A2 molecules. In addition, we conducted the MAGE-A3-Mp4 epitope-specific TCR repertoire seqencing, and systematically screened candidate MAGE-A3 antigen-specific TCRs with high specificity by co-considering T cell cellular phenotype and clonotype expansion. Furthermore, the tumor-reactive TCRs with killing potency was screened and verified, which might benefit personalized TCR-T cell therapy and prediction of immunotherapy response in NSCLC.

The accumulation of variable genetic alterations and the loss of normal cellular regulatory processes result in the expression of neoantigens, differentiation antigens, or CTAs 49. Tumor-specific peptide-MHC-I complexes can be recognized by CD8+ T cells, which are the primary mediators of anticancer immunity 50. However, they rarely provided protective immunity due to the insufficient production of antigen-specific T cells or dysfunction of tumor specific T cells 5. Thus, modulation of the CD8+ T cell response has been a central focus of immunotherapy to treat cancer, such as TCR-T cell therapy. A latest study 15 described a phase 1 TCR-T therapy targeting MAGE-A4 could be a promising treatment for HLA-A2+ patients with MAGE-A4-expressing solid tumors.

MAGE-A3 is a CTA characterized by tumor restriction and high immunogenicity 51. It has been reported that MAGE-A3 is highly expressed in many cancers, such as melanomas and lung cancer 11, 52. In our study, we found MAGE-A3 protein was also a potential immunotherapy target for the treatment of NSCLC. MAGE-A3 immunotherapeutic is well tolerated and induces MAGE-A3 specific immune responses in phase I/II studies 53, 54. However, a multicenter phase 3 study indicated that 13 adjuvant treatment with the recombinant MAGE-A3 protein AS15 did not increase disease-free survival in surgically resected NSCLC patients that were MAGE-A3-positive. Therefore, novel strategy of immunotherapy targeting MAGE-A3 is still needed.

T cells expressing tumor antigen-specific TCR can recognize the HLA-peptide complex on the surface of tumor cells, which in turn causes the immune effect of T cells to kill tumor cells 55. We enriched the MAGE-A3-Mp4 epitope-specific CD8+ T cells by tetramer for scRNA-seq and scTCR-seq to generate high-quality transcriptome and TCR library data. The MAGE-A3 epitope-specific T cell transcriptome and TCR repertoire data is a critical resource to screen T cells with TCRs for high antitumoral immune response in NSCLC.

One advantage of TCR-T is the natural sensitivity of TCRs to the very low antigen densities on tumors. However, a drawback is that most endogenous tumor-specific TCRs have low affinity for tumor-associated pMHCs that only weakly activate the TCR-T cells they bind to 8. The strategy to improving this was to increase the affinity of the TCR. However, the affinity matured TCRs recognizing MAGE-A3 has reported substantial off-target toxicities in clinical trials because of the high affinity 19, 21. Thus, the key work is to identify tumor-reactive TCRs with appropriate affinity to enhance their killing potency and avoid off-target toxicities. In the present study, we combined the MAGE-A3 epitope specific TCR repertoire and artificial intelligence analysis pMTnet and tessa to screen TCR for further tests. J76 cells with the expression of 5 candidate TCRs (TCR-1, TCR-2, TCR-12, TCR-103 and TCR-207) recognized the MAGE-A3 tumor antigen on HLA-A2 and led to target cell lysis. In addition, the TCRmodel2 docking prediction results showed that HLA-A2-MAGE-A3-Mp4-TCR12 pHMC-TCR complex had the best binding stability and there was low risk of cross-reaction with a muscle protein, Titin 21 or MAGE-A12 expressed in human brain 19, which were previously reported for off-target toxicity. Overall, these results suggest our study represented a plausible approach toward the development of TCR-T cell immunotherapy targeting MAGE-A3-driven NSCLC and other solid tumor.

ICB immunotherapy can provide long-lasting clinical responses and the 5-year overall survival rate currently exceed 25% among patients whose tumors have high PD-L1 expression 56-58. However, only 20-30% patients can benefit from PD-1 ICB, and adaptive resistance often happens after initial response. The presence of highly polymorphic TCR in the individual determines the adaptive immune system responds to immunotherapy and recognizes tumor antigens 50. However, insufficient anti-tumor T-cell generation and inadequate anti-tumor TCR function are the main reasons of resistance to ICB 58. Characterization of the TCR repertoire in terms of clones, diversity, and antigen specificity through the CDR3 hypervariable region, seems to be a promising approach to predict the clinical responses of the patients to ICB immunotherapy 59. By comparing TCR sequences from lung cancer patients treated with ICB therapy with the MAGE-A3 antigen-specific TCRs, we found that the matching TCR clonotypes increased after ICB treatment, and patients with TCR repertoire that were more similar to the candidate TCRs would have better response to ICB therapy. These results implies that MAGE-A3-specific TCRs might play an important role in the anti-tumor immune responses in lung cancer.

Previously, two HLA-A2-restricted peptides of MAGE-A3: 112-120 and MAGE-A3: 271-279, respectively, were used to immune mice and screen MAGE-A3-specific TCRs 32. However, the TCR against MAGE-A3: 112-120 was also recognized by MAGE-A12 peptide epitopes, and further TCR gene-modified MAGE-A3 TCR-T clinical trials caused neurologic toxicity 19. In addition, MAGE-A3-specific TCRs in the context of HLA-DPB1*04:01 were isolated and identified from a regulatory T-cell clone (6F9), which was generated from the peripheral blood of 2 melanoma patients after MAGE-A3 vaccination 9. However, systematic analysis of T cell immune responses to MAGE-A3 antigen and corresponding antigen-specific TCR in human in the context of HLA-A2 is still lacking. In this study, we re-confirmed Mp1 (MAGE-A3: 112-120) and Mp2 (MAGE-A3: 271-279) were immunogenic epitopes. While Mp4 (MAGE-A3: 160-169) could induce much higher antitumoral immune response. We also identified the candidate TCRs specific to MAGE-A3-Mp4. Based on these findings, we will further evaluate the killing effect of MAGE-A3-specific TCR-T using primary T cells *in vitro* and *in vivo* before initiating a clinical trial. By amplifying the naturally occurring antitumor immunity in patients, the MAGE-A3-specific TCR-T cell therapy may generate efficient and safe antitumor immune response and provide an option to patients resistant to current immunotherapies.

## Supplementary Material

Supplementary figure legends and table legends.

Supplementary figures and tables.
