# Supplementary material for: Global analysis of HLA-A2 restricted MAGE-A3 tumor antigen epitopes and corresponding TCRs in non-small cell lung cancer
Source: Theranostics. 2023 Aug 6;13(13):4449–68. doi: 10.7150/thno.84710 (PMC10465222; doi:10.7150/thno.84710)
Supplement: Supplementary file 1 — Supplementary figure legends and table legends. [file thnov13p4449s1.pdf]

Supplementary Figures

Supplementary Figure Legends

Figure S1. Immunohistochemistry of MAGE-A3 protein in LUAD and LUSC.

(A) Scan image of LUAD (left) and LUSC (right) tumor tissue and corresponding paracancerous area microarray after immunohistochemical staining of MAGE-A3 protein. Tumor tissue (odd number columns) and corresponding paracancerous area (even number columns) are placed adjacently.

(B) Bar charts summarizing the IRS of MAGE-A3 in LUAD (left) and LUSC (right) from different pathological groups. Data are shown as mean ± SD. **$P < 0.01$. Each dot represents a single sample. Statistical significance was determined by one-sided t-test.

Figure S2. FACS gating strategies for specific CD8+ T cells, and T2 cell proliferation inhibition assay.

(A) Flow cytometry gating strategy for MAGE-A3 epitope-specific CD8+ T, GZMB+ CD8+ T and IFN-γ+ CD8+ T cells, respectively.

(B) T2 cell proliferation inhibition assay. Left: Representative FACS result; Right: Corresponding summary statistics of Left. Data are shown as mean ± SD. ***$P < 0.001$. Statistical significance was determined by one-sided t-test.

Figure S3. Sorting of MAGE-A3-Mp4 epitope-specific CD8+ T cells for single cell sequencing.

(A) Flow cytometry gating strategy for MAGE-A3-Mp4 epitope specific CD8+ T cells. CD8+ T cells from HLA-A2+ healthy donors were co-cultivated with peptide-loaded T2 cells. Sorted Mp4 Tetramer+ CD8+ T cells were subject for scRNA-seq and scTCR-seq analysis on day 7.

(B) FACS plots of pre-sorting of MAGE-A3-Mp4 epitope-specific CD8+ T cells from 2 healthy donors for scRNA-seq and scTCR-seq analysis.

(C) FACS plots of post-sorting of MAGE-A3-Mp4 epitope-specific CD8+ T cells

from 2 healthy donors for scRNA-seq and scTCR-seq analysis.

Figure S4. scRNA-seq data quality assessment and visualization of selected marker gene expression.

(A) Violin plots of number of unique genes (left), number of expressed genes (middle) and percentage of mitochondria genes (right) detected by scRNA-seq data from each donor. The horizontal dashed lines in the left panel mark 200 and 6,000, respectively. The horizontal dashed line in the right panel marks 10%. Mt: mitochondria.

(B) PCA visualization of scRNA-seq data for CD8+ T cells specific to MAGE-A3-Mp4 epitope from each donor.

(C) UMAP visualization of scRNA-seq data with selected marker gene expression projection.

Figure S5. Single-cell transcriptome analysis of CD8+ T cells from healthy adults without stimulation, and single-cell TCR landscape of CD8+ T cells specific to MAGE-A3-Mp4 from each donor.

(A) UMAP visualization of CD8+ T cell scRNA-seq data from two HLA-A2 healthy young adults. The identified cell clusters are labeled with distinct colors.

(B) Dot plot of marker genes for each T cell subtypes in (A). Color-scale shows the average normalized expression of marker genes in each subtype, and dot size indicates the percentage of cells within each cell cluster expressing the marker gene.

(C) Pie chart summarizing the proportion of each CD8+ T cell subtype in (A).

(D) Same UMAP visualization as Figure 4C, but with TCR sequence detection information (left), TCR clonotype expansion (clonotype frequency > 1) information (middle), and top 5 most frequent TCR clonotype information (right) projected on and split by each donor.

(E) Same UMAP visualization as Figure 4C, but with top 5 most frequent TCR clonotype information of P1 (left) and P2 (right) projected on and split by each donor.

Figure S6. Screening of HLA-A2+MAGE-A3+ cancer cell lines.

(A) FACS plots of J76 cells expressing the candidate TCRs.

(B) FACS plots of HLA-A2 staining in selected cancer cell lines. HLA-A2+ cell lines are shown in red.

(C) Western-blot image of MAGE-A3 protein expression level in melanoma, lung and liver cancer cell lines.

Supplementary Tables

Table S1: Participants information of NSCLC cohort, Related to Figure 3.

Table S2: Clinical features of the LUAD tissue microarray, Related to Figure 1.

Table S3: Clinical features of the LUSC tissue microarray, Related to Figure 1.

Table S4: Top 10 marker genes of CD8+ T cell subtypes from scRNA-Seq, Related to Figure 4.

Table S5: List of gene panels for calculating functional scores for CD8+ T cell clusters defined by scRNA-seq data, Related to Figure 4.
