# Supplementary figures and images for: Global analysis of HLA-A2 restricted MAGE-A3 tumor antigen epitopes and corresponding TCRs in non-small cell lung cancer
Source: Theranostics. 2023 Aug 6;13(13):4449–68. doi: 10.7150/thno.84710 (PMC10465222; doi:10.7150/thno.84710)

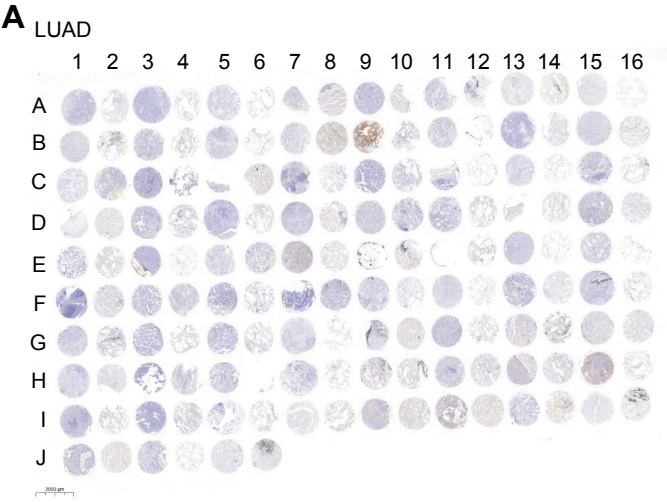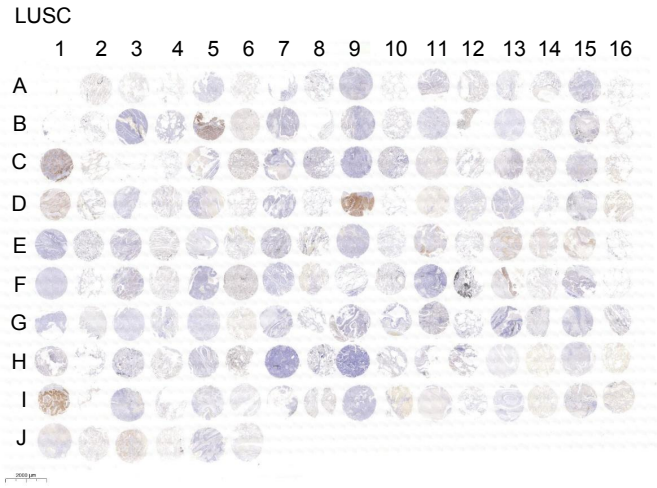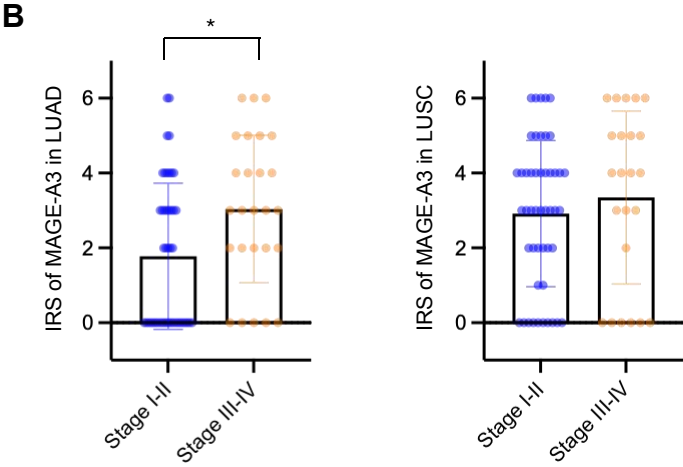

Supplement: Supplementary file 2 — Supplementary figures and tables. [file thnov13p4449s2.zip › Supplementary files/Figure S1.pdf]

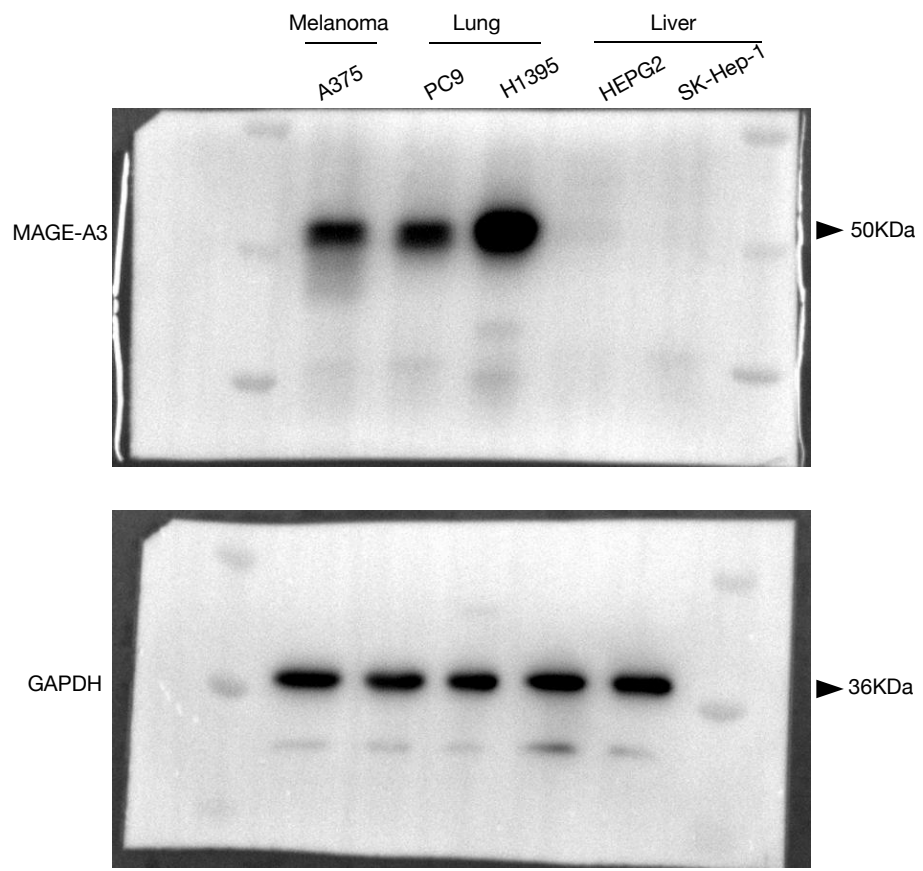

Supplement: Supplementary file 2 — Supplementary figures and tables. [file thnov13p4449s2.zip › Supplementary files/Raw imaging for Western blots.pdf]

**A**

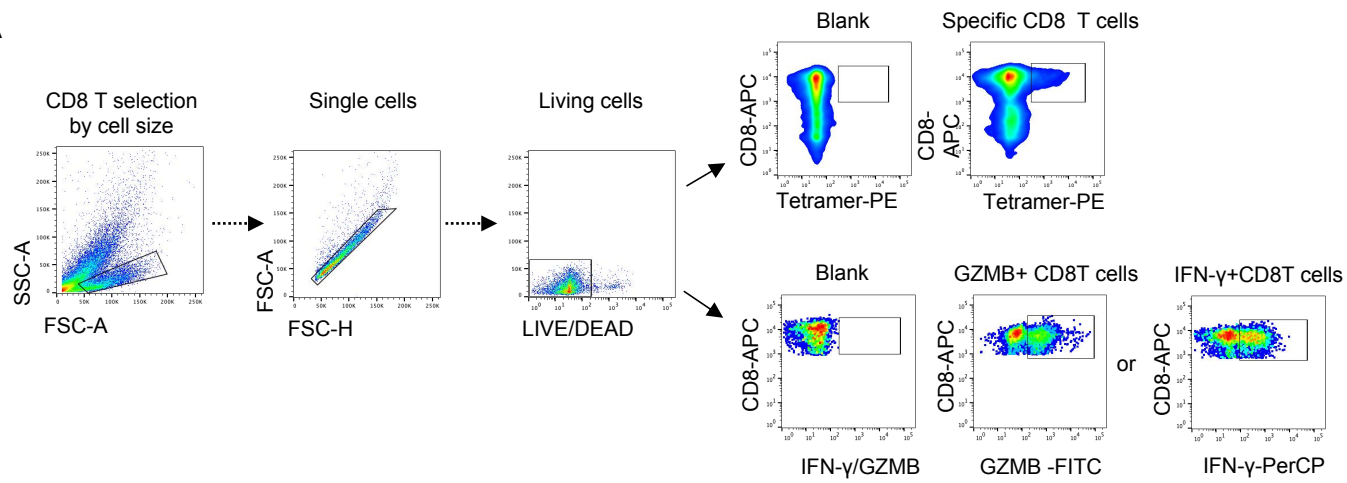

**B**

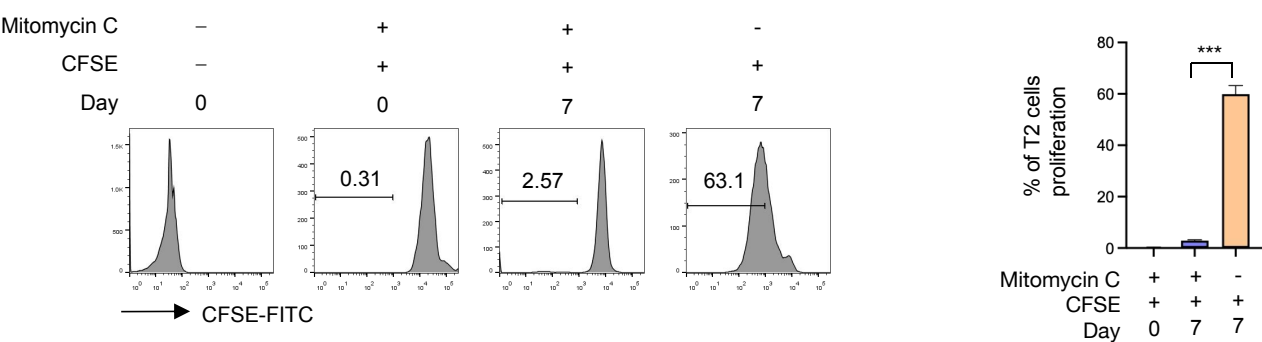

Supplement: Supplementary file 2 — Supplementary figures and tables. [file thnov13p4449s2.zip › Supplementary files/Figure S2.pdf]

**A**

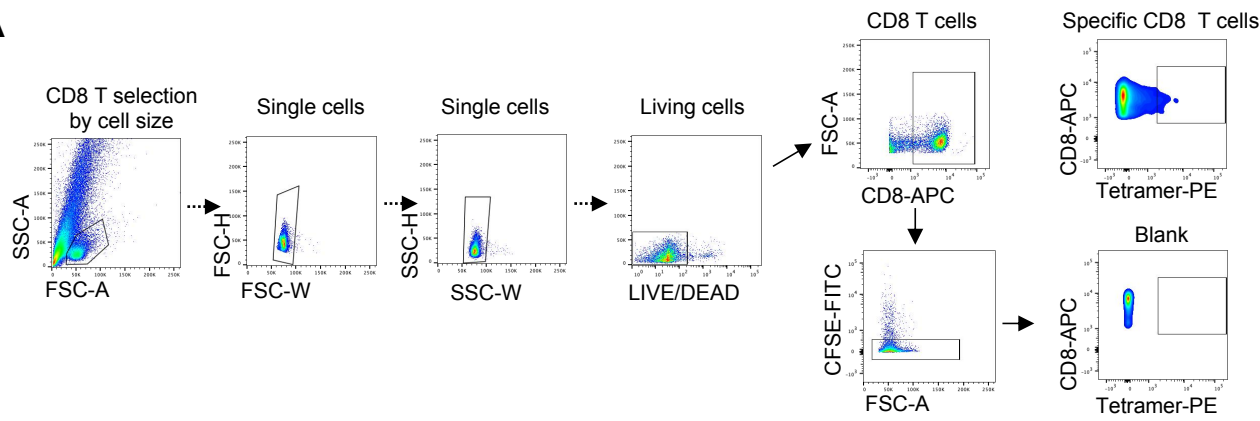

**B**

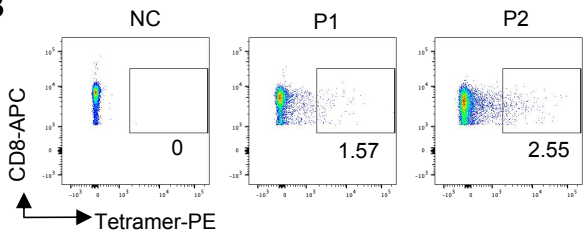

**C**

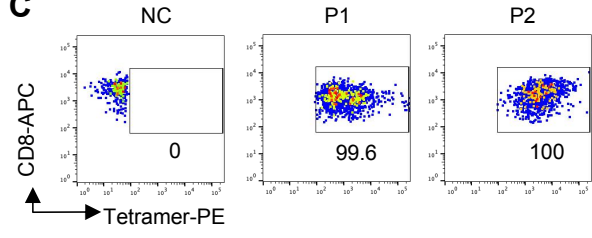

Supplement: Supplementary file 2 — Supplementary figures and tables. [file thnov13p4449s2.zip › Supplementary files/Figure S3.pdf]

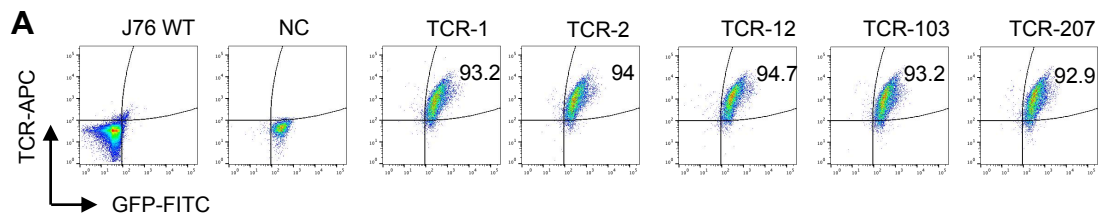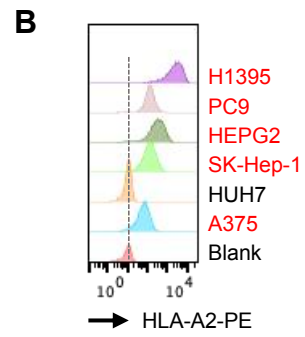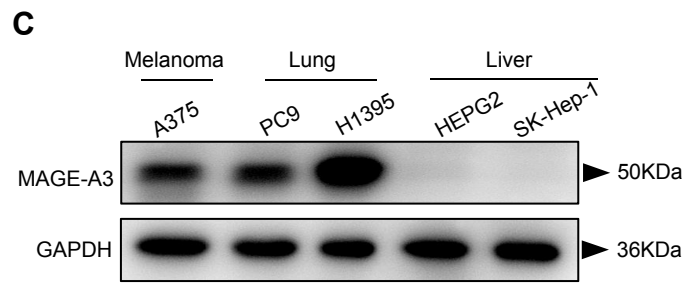

Supplement: Supplementary file 2 — Supplementary figures and tables. [file thnov13p4449s2.zip › Supplementary files/Figure S6.pdf]

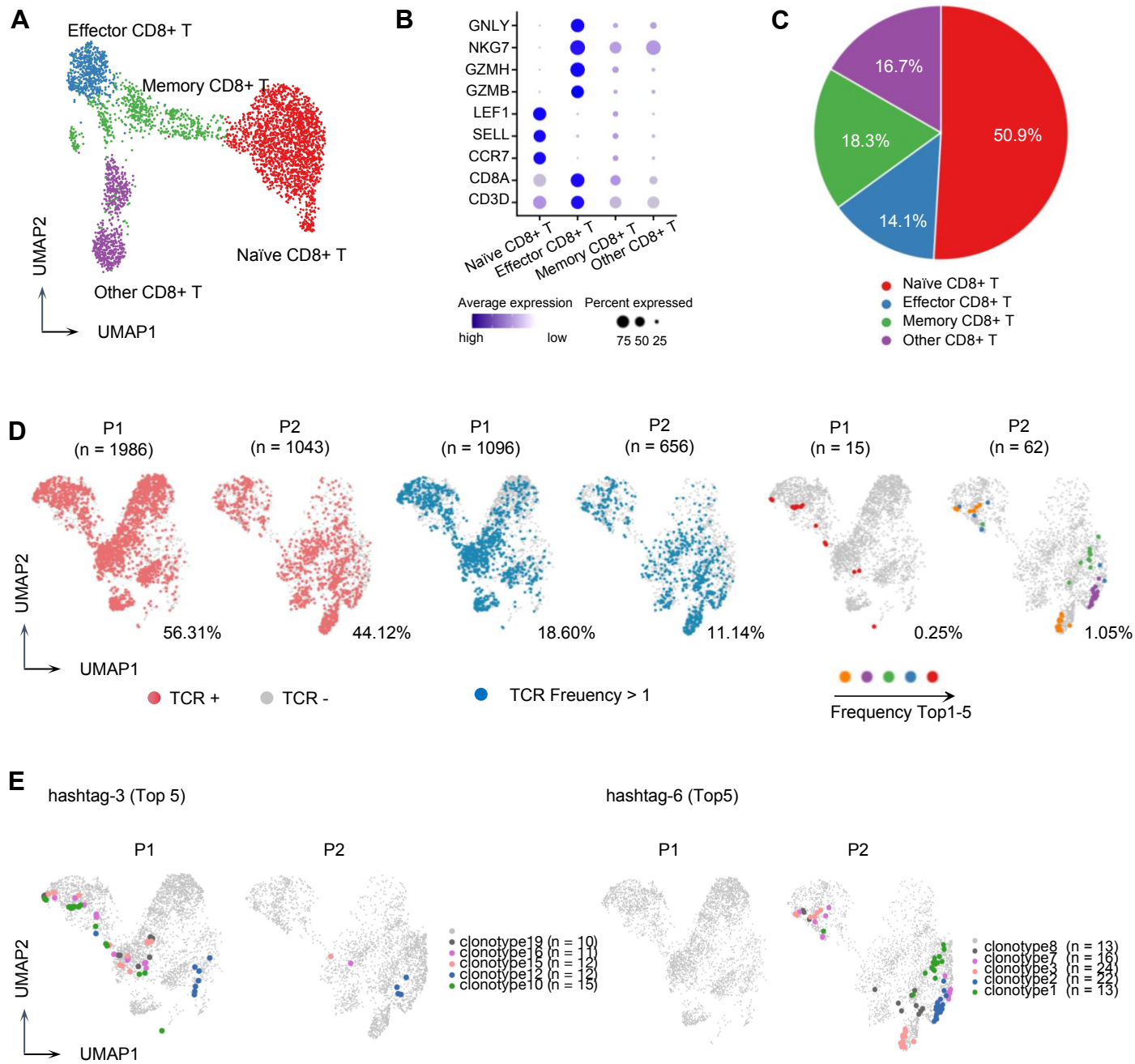

Supplement: Supplementary file 2 — Supplementary figures and tables. [file thnov13p4449s2.zip › Supplementary files/Figure S5.pdf]
