# Supplementary material for: Global analysis of HLA-A2 restricted MAGE-A3 tumor antigen epitopes and corresponding TCRs in non-small cell lung cancer
Source: Theranostics. 2023 Aug 6;13(13):4449–68. doi: 10.7150/thno.84710 (PMC10465222; doi:10.7150/thno.84710)
Supplement: Supplementary file 2 — Supplementary figures and tables. [file Figure S4.pdf › Supplementary files/Figure S4.pdf]

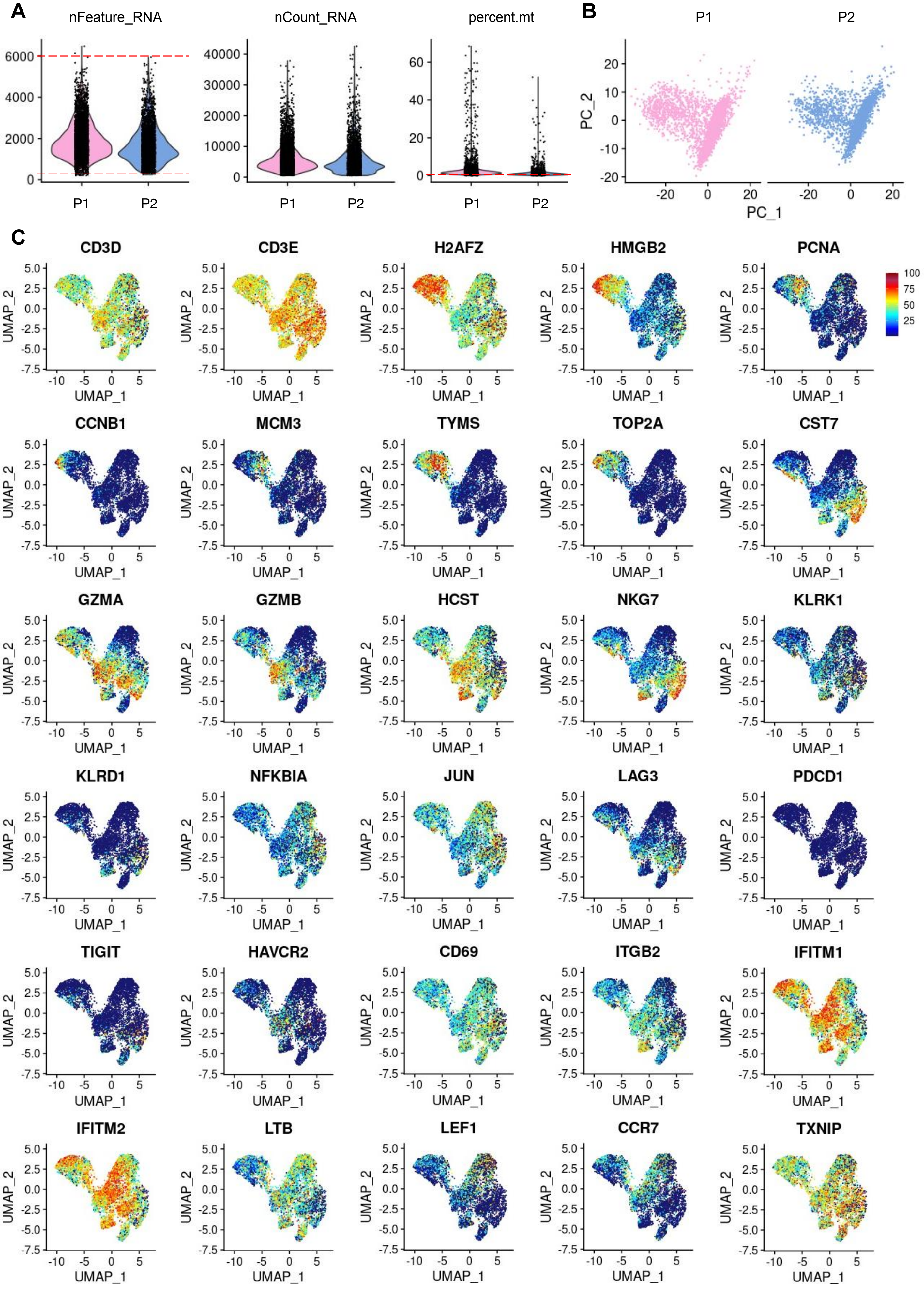

A
nFeature_RNA
nCount_RNA
percent.mt
P1
P2
B
P1
P2
PC_1
PC_2
C
CD3D
CD3E
H2AFZ
HMGB2
PCNA
CCNB1
MCM3
TYMS
TOP2A
CST7
GZMA
GZMB
HCST
NKG7
KLRK1
KLRD1
NFKBIA
JUN
LAG3
PDCD1
TIGIT
HAVCR2
CD69
ITGB2
IFITM1
IFITM2
LTB
LEF1
CCR7
TXNIP
UMAP_1
UMAP_2
100
75
50
25
